# Thermal and Magnetic Dual-Responsive Catheter-Assisted Shape Memory Microrobots for Multistage Vascular Embolization

**DOI:** 10.34133/research.0339

**Published:** 2024-03-26

**Authors:** Qianbi Peng, Shu Wang, Jianguo Han, Chenyang Huang, Hengyuan Yu, Dong Li, Ming Qiu, Si Cheng, Chong Wu, Mingxue Cai, Shixiong Fu, Binghan Chen, Xinyu Wu, Shiwei Du, Tiantian Xu

**Affiliations:** ^1^ Guangdong Provincial Key Lab of Robotics and Intelligent Systems, Shenzhen Institute of Advanced Technology, Chinese Academy of Sciences, Shenzhen, China.; ^2^ University of Chinese Academy of Sciences, Beijing, China.; ^3^ Department of Neurosurgery, South China Hospital, Medical School, Shenzhen University, Shenzhen, China.; ^4^ The Key Laboratory of Biomedical Imaging Science and System, Shenzhen Institute of Advanced Technology, Chinese Academy of Sciences, Shenzhen, China.

## Abstract

Catheters navigating through complex vessels, such as sharp turns or multiple U-turns, remain challenging for vascular embolization. Here, we propose a novel multistage vascular embolization strategy for hard-to-reach vessels that releases untethered swimming shape-memory magnetic microrobots (SMMs) from the prior catheter to the vessel bifurcation. SMMs, made of organo-gel with magnetic particles, ensure biocompatibility, radiopacity, thrombosis, and fast thermal and magnetic responses. An SMM is initially a linear shape with a 0.5-mm diameter at 20 °C inserted in a catheter. It transforms into a predetermined helix within 2 s at 38 °C blood temperature after being pushed out of the catheter into the blood. SMMs enable agile swimming in confined and tortuous vessels and can swim upstream using helical propulsion with rotating magnetic fields. Moreover, we validated this multistage vascular embolization in living rabbits, completing 100-cm travel and renal artery embolization in 2 min. After 4 weeks, the SMMs maintained the embolic position, and the kidney volume decreased by 36%.

## Introduction

**Fig. 1.**
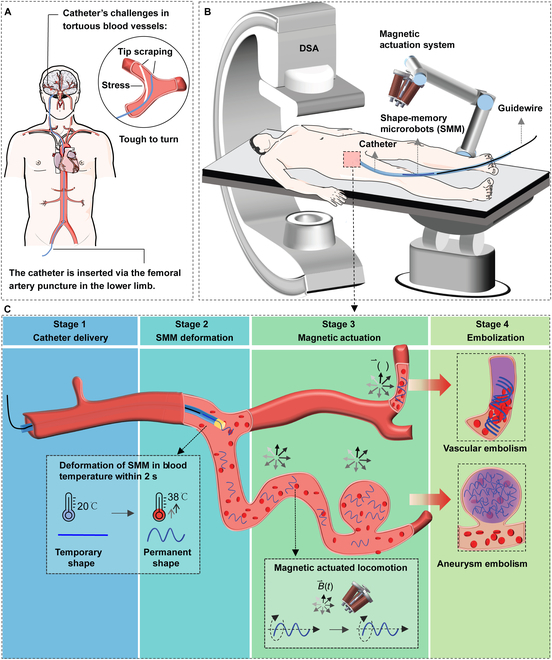
Schematic representation of the proposed multistage strategy for targeted embolization. (A) Overview of the interventional procedure, illustrating the path and the challenges of the catheter in the vascular system. (B) Schematic diagram of our integrated platform for assisting the clinical application of SMMs: DSA (digital subtraction angiography), a magnetic actuation system, a catheter, and a guidewire. (C) Four steps of the multistage embolization strategy. Step 1—Catheter Delivery: The catheter is entered via the femoral artery puncture, advancing through a relatively straight aorta to the vicinity of the tortuous vessels. Linear SMMs are inserted from the catheter end and pushed by the guidewire toward the catheter head. Step 2—SMM Deformation: Within 2 s of being pushed out of the catheter into the blood, linear SMMs are transformed into helical shapes due to blood temperature (38 °C). Step 3—Magnetic Actuation: Helical SMMs swim through sharp turns or multiple U-turns to the embolization site with a rotating magnetic field. Step 4—Embolization: Embolization on the vessel or aneurysm is achieved at the target site.

Accessing the vascular system using catheters has revolutionized minimally invasive diagnostic and therapeutic procedures, providing numerous clinical benefits [1,2]. However, the distal vascular routes pose marked challenges for safe catheter access due to various factors, including longer access routes, vessel tortuosity, and delicate blood vessel walls [3,4]. As illustrated in Fig. 1A, catheters are inserted through the femoral artery in the lower extremity during interventional procedures. These catheters navigate from the relatively straightforward aorta to the intricate and tortuous distal branch vessels. This complicated navigation process requires the continuous attention and skill of the surgeon. Such a process carries the risk of vessel wall damage, including stress and tip scraping (Fig. 1A). It also prolongs the procedure and increases the utilization of contrast agents, thereby escalating the procedural risks [5–9]. These challenges lie in accessing the distal cortical arteries, which are crucial for treating various lesions and diseases in that region, including aneurysms [10–12], arteriovenous malformations [13,14], liver tumors [15,16], vascular tumors [17], and bleeding control [18–20]. Please refer to Table S1 for current limitations for these diseases. Hence, it is difficult to navigate and push the catheter into tortuous vessels without causing injuries by either manual or robotic insertion [21,22]. Additionally, the size of the inner diameter of the catheter limits the substances or devices that can be transported through it. Delivering therapeutic agents such as coils, stents, microspheres, gelatin sponge particles, and polymeric materials becomes challenging [23]. Therefore, there is a need for more effective medical tools for minimally invasive procedures in hard-to-reach regions.

In recent years, substantial efforts have been made to develop micro/nanomachines (MNMs) [24–27]. These MNMs, with their tetherless nature and small dimensions, can access hard-to-reach sites in the body that have shown great potential in medical applications, particularly in navigating confined spaces, delivering cargo, and performing targeted tasks [28–34]. MNMs can be remotely controlled and actuated using various methods such as magnetic, light, and chemical actuation [35–40]. Magnetic actuation is particularly attractive because of the potential to wirelessly control MNM locomotion speed and direction while guaranteeing harmless interaction with cells and tissues [36,41–43]. Helical micromachines (HMMs) are typical representatives of magnetic-propelled MNMs, which mimic the motion of bacterial flagella. Compared with other MNMs that rely on alternating magnetic fields or magnetic field gradients, HMMs offer safety and high-accuracy movement due to an efficient torque-driven strategy under low-strength rotating magnetic fields [41,44]. Therefore, magnetic HMMs have received increasing interest in biomedical applications. However, previous designs have not met all the requirements for clinic operation. Several crucial aspects remain to be addressed in future research, including integrating functions, real-time positioning, developing magnetic control platforms with larger workspaces, and tracking in vivo applications of HMMs [45,46]. Additionally, the applications of HMMs in vascular treatments have mostly been limited to simple in vitro studies, and there is a need for more progress in practical applications [47]. Therefore, to enhance clinical applications, it is essential to integrate time-efficient therapy delivery, modern medical imaging, and treatment effectiveness within HMMs.

To address the challenges mentioned above, we propose a novel multistage vascular embolization strategy utilizing the untethered swimming shape-memory magnetic microrobots (SMMs) from the prior catheter to the vessel bifurcation, which provides time-efficient delivery and access to hard-to-reach vessels for embolization in vivo. SMMs, made of organo-gel with 20 wt% magnetic particles, ensure biocompatibility by histopathological findings, radiopacity in x-ray imaging, thrombosis in animal experiments, high shape memory performance in DMA test, and fast thermal and magnetic responses. An SMM is initially a linear shape with a 0.5-mm diameter at 20 °C inserted in a catheter. It transforms into a predetermined helical shape within 2 s at 38 °C blood temperature after being pushed out of the catheter into the blood. Helical SMMs enable agile swimming in confined and tortuous vessels with rotating magnetic fields. Some shape memory gels have been developed for vascular and aneurysm embolization [48,49]. However, limited literature exists on shape memory gels that can rapidly recover their memorized shape at blood temperature and be driven wirelessly [50] (see Table S2). We explored the multimodal locomotion adaptation of SMMs in a carotid artery model with gradients, swimming upstream against water flow and navigating over long distances in a human-sized model. Our integrated platform combines digital subtraction angiography (DSA), a magnetic actuation system (MAS), a catheter, and a guidewire, ensuring rapid, precise, real-time deployment and delivery of SMMs inside the body (Fig. 1B). Figure 1C illustrates 4 sequential steps of the multistage embolization strategy using catheter-assisted SMMs, including catheter delivery, SMM deformation, magnetic guidance, and embolization. Firstly, the catheter is inserted via the femoral artery puncture point of the lower limb. It advances through a relatively straight aorta to the vicinity of the tortuous vessels. Then, linear SMMs at 20 °C are inserted from the catheter’s end and pushed by the guidewire toward the catheter head. Secondly, linear SMMs transform into predetermined helical SMMs within 2 s at 38 °C after being pushed out of the catheter into the blood. Thirdly, helical SMMs can swim agilely in a rotating magnetic field and are capable of passing through sharp turns or continuous U-turns to the target embolization site. Finally, the helical SMMs complete the embolization of the vessel or aneurysm. We validated the whole multistage embolization in living rabbits, achieving downstream and upstream locomotion of the SMM in blood flow, completing 100-cm travel and renal artery embolization in 2 min. Four weeks postoperatively, the embolized kidney volume is reduced by 36%, and SMMs remain complete embolization of the vessel without displacement.

## Results

### Composition, fabrication, and material properties of SMMs

**Fig. 2.**
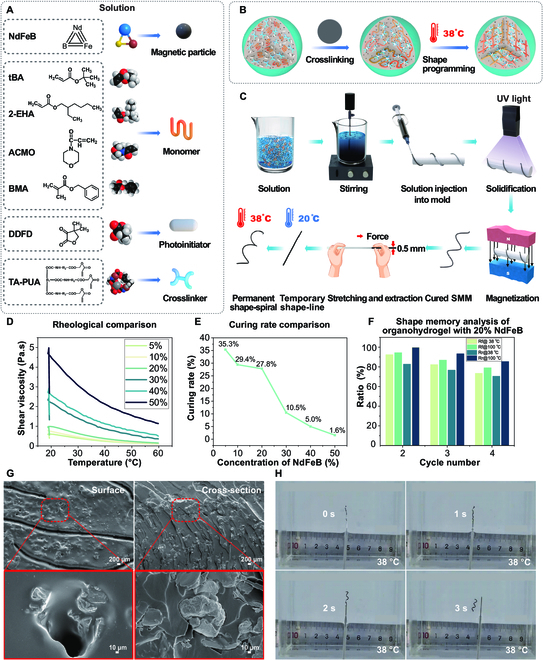
Composition, fabrication, and material properties of the shape-memory magnetic microrobots (SMMs). (A) Compositions of the SMM. (B) Visualization of the internal structure transformation of the SMM. (C) Stepwise fabrication process of the SMM. (D) Rheological analysis of SMMs with varying concentrations of NdFeB magnetic particles at different temperatures. The solution with 20 wt% magnetic particles exhibited a suitable shear viscosity of 608 to 982 mPa.s at 20 to 30 °C. (E) The curing rate of the solution decreased with increasing concentration of NdFeB magnetic particles. The curing rate of solutions with 5 to 20 wt% magnetic particle concentration was higher than those with 30 to 50 wt%. (F) Shape memory analysis of the SMM with 20 wt% magnetic particle concentration. The shape fixing ratio (Rf) and the shape recovery ratio (Rr) remained above 70% during a 3-h testing period at both 38 °C and 100 °C. (G) SEM image shows the dense internal structure of the SMM with 20% magnetic particle concentration. (H) Snapshot from the deformation video of the SMM (Movie S1).

The SMMs are fabricated using the solution described in Fig. 2A, which includes the magnetic particle NdFeB; the monomers tert-butyl acrylate (tBA), 2-ethylhexyl acrylate (2-EHA), 4-acryloylmorpholine (ACMO), and benzyl methacrylate (BMA); the edible photoinitiator 4,4-dimethyldihydrofuran-2,3-dione (DDFD); and the crosslinker trifunctional aliphatic urethane acrylate (TAPUA-B368). Detailed chemical information can be found in Materials and Methods. Figure 2B shows that the solution forms a dense organo-gel network upon crosslinking. The relaxed segments in the organo-gel will transform into stretched segments at 38 °C, and the 4-dimensional shape memory effect depends on this state transition in response to thermal stimulation. Figure 2C illustrates the fabrication process of the SMM. The solution is stirred with a magnetic stirring machine and injected into a transparent silicone tube with a 0.5-mm inner diameter. The silicone tube is then twisted around a threaded mold, and after being exposed to ultraviolet (UV) light for 30 s and magnetized for 2 s, a helical SMM inside the silicone tube is obtained. To temporarily fix the linear shape of the SMM, the silicone tube is straightened at 20 °C, and the SMM inside is withdrawn with a fixed temporary linear shape. When the temperature reaches 38 °C, the SMM deforms from a temporary linear shape to a permanent helical shape within 2 s due to the memory properties of the material. The detailed fabrication process can be found in Materials and Methods.

The shear viscosity increased with increasing concentration of NdFeB in Fig. 2D. The solution with 20 wt% NdFeB demonstrated a shear viscosity of 608 to 982 mPa.s from 20 to 30 °C, which facilitated injection into the mold as liquid at room temperature. The curing rate of the solution decreases as NdFeB concentration increases. A decrease in curing rate is observed between 20 wt% and 30 wt% magnetic particle concentration. Solutions containing 5 to 20 wt% magnetic particles have higher curing rates than those containing 30 to 50 wt% magnetic particles (Fig. 2E). The solution’s curing rates were calculated from the Fourier transform infrared spectroscopy (FTIR) spectra in Figs. S1 and S2. The solution with 20 wt% magnetic particles maintained its shape memory performance for up to 4 DMA cycles (Fig. 2F). Both the shape fixing ratio (Rf) and the shape recovery ratio (Rr) remained above 70% during a 3-h testing period at both 38 °C and 100 °C. Higher temperatures resulted in increased ratios. In the second cycle of the DMA test at 38 °C, Rf and Rr are 92.46% and 82.79%, respectively (as described in Fig. S3). At a blood temperature of 38 °C, the material’s elastic modulus is 31 MPa, and tensile strength is 0.68 MPa (as described in Fig. S4). The elastic modulus and tensile strength of the renal artery range from a few hundred kilopascals to a few megapascals. The SMMs have a tensile strength similar to that of an arterial wall, and its modulus of elasticity is 10 times greater than the maximum modulus of elasticity of an artery. As a result, the SMM is soft and flexible enough relative to the arterial vessel wall to effectively avoid damage to the vessel wall during the rotational process. The scanning electron micrograph (SEM) in Fig. 2G presents the dense internal structure of SMM with 20 wt% concentration of NdFeB. Figure 2H shows the transformation of the SMM from a linear shape to a helical shape at 38 °C after being pushed out of the catheter by the guidewire (as shown in Movie S1).

To conclude, the SMM is prepared from a specified solution, which is able to rapidly transform from a linear shape to a helical shape at 38 °C. At room temperature from 20 to 30 °C, the solution containing 20 wt% magnetic particles exhibited a suitable shear viscosity ranging from 608 to 982 mPa·s. When exposed to 38 °C, it demonstrated a high shape memory performance, with a shape fixity ratio of 92.46% and a shape recovery ratio of 82.79%.

### Design, optimization, and evaluation of the kinematic performance of SMMs

**Fig. 3.**
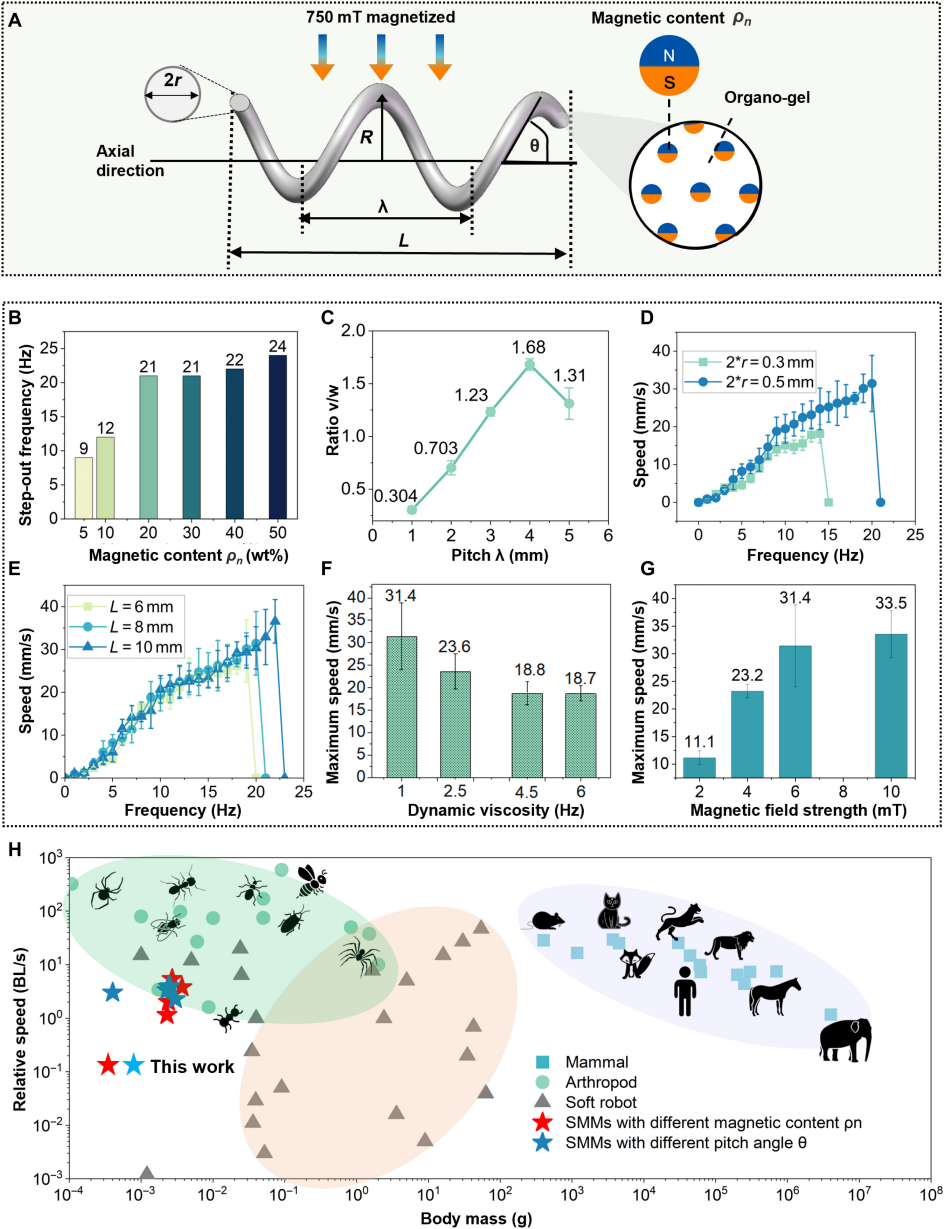
Optimization of the SMM’s kinematic performance by the design of magnetic particle concentration, shape geometry parameters, and comparison of mass–speed relationship among different species. (A) Schematic shows key geometric parameters of the helical structure, including wire radius (*r*), helical radius (*R*), pitch (λ), length (*L*), and angle (θ), alongside the magnetic particle concentration (ρn). Use a 6-mT uniform magnetic field for parametric optimization. (B) Step-out frequencies ω_step-out_ increased with increasing magnetic particle concentration, stabilizing between 20 wt% and 50 wt%, with both 20% and 30% showing identical values. (C) An SMM with a pitch of 4 mm exhibited a higher speed–frequency ratio *v*/ω than other pitch lengths. (D) An SMM with a wire diameter of 0.5 mm exhibited higher *v*/ω and ω_step-out_ than a 0.3-mm diameter. (E) SMMs with different total length (*L*) showed approximate *v*/ω and ω_step-out_. (F) The maximum speed of SMM decreased with increasing glycerol-water viscosity. (G) The maximum speed of SMM increased with increasing magnetic field strength, plateauing at around 8 mT. (H) Speed–mass relationships differ between arthropods (green), robots (gray), mammals (blue), and SMMs (stars). In arthropods and mammals, relative speed is inversely proportional to body mass, whereas soft robots show the opposite trend. SMMs (red stars for magnetic content ranging from 5% to 50%; blue stars for pitch length from 1 mm to 5 mm) showed a nonlinear speed–mass relationship. Optimal design at 0.0027 g with 5.08 BL/s (detailed data are shown in Table S3).

Magnetic particle concentration and shape geometry parameters play a crucial role in swimming helical microrobots. SMMs are characterized by magnetic particle concentration (ρn) in weight ratio and a set of geometric parameters, including the radius of wire (*r*), the radius of the helix (*R*), the pitch of the helix (λ), the number of turns (*n*), overall length (*L* = λ × *n*), and the helix angle (θ = arctan(2π*R*/λ)) (Fig. 3A). Optical pictures of helical SMMs with different geometrical parameters are shown in Fig. S5.

The SMMs are magnetized at 750 mT perpendicular to the axial direction, followed by testing their axial velocity with a uniform rotating magnetic field. Purcell shows that the linear relationship between the forward velocity *v*, rotational speed ω, external force *F*, and torque τ on a helix can be expressed in matrix form [51,52].

Fτ=ABBCvw (1)

*A*, *B*, and *C* are coefficients that depend on the fluid’s viscosity and the helix’s shape geometry. The speed-to-frequency ratio *v*/ω represents the sensitivity of helical microrobots to magnetic fields. Step-out frequency ω_step-out_ determines the maximum frequency to which the helix can respond, which influences the achievable maximum velocity. Therefore, *v*/ω and ω_step-out_ are 2 critical parameters characterizing the kinematic motion of the helix (see Text S1 for details).

Figure 3B illustrates that the ω_step-out_ increased with the concentration of magnetic particles from 0 to 20 wt% and saturated at 20 wt%. Radiographic visibility of SMMs with different magnetic particle concentrations showed the same results (shown in Fig. S6). Thus, the optimal concentration is 20 wt%. Figure 3C shows that *v*/ω of the SMM peaked at pitch = 4 mm. According to the above conversion formula, when using an SMM with a pitch length of 4 mm, the corresponding pitch angle was 63°. The SMM with 0.5 mm wire diameter had higher *v*/ω and ω_step-out_ than the SMM with 0.3 mm wire diameter (Fig. 3D). Different total length (*L*) of the SMM showed approximate *v*/ω and ω_step-out_ (Fig. 3E). Thereby, the helical SMM with magnetic particle concentration ρn = 20 wt%, pitch angle θ = 63°, wire diameter *r* = 0.5 mm, and total length *L* = 8 mm is selected as the best design for the following experiments. Figure 3F demonstrates that the SMM’s maximum speed decreases with increasing viscosity of the water–glycerol mixture solution. The maximum velocity increased as the magnetic field strength increased (Fig. 3G). Figure S7 displays the complete data for Fig. 3B to G. Speed–mass relationships for some arthropods (green), soft robots and actuators (gray), mammals (blue), and SMMs (red and deep blue stars) are shown in Fig. 3H [53,54]. Arthropods and mammals show that the speeds decrease as their body masses increase. Soft robots and actuators show that the speeds increase as the body mass increases. SMM’s maximum speed will first be increased and then decreased, reaching a peak speed of 5.42 BL/s at a weight of 0.0027 g (detailed data are shown in Table S3).

To conclude, the 20 wt% magnetic particle concentration of the SMM was optimal in terms of magnetic kinematic response and x-ray imaging visibility (Fig. S6). The SMM with a pitch angle of 63° and a wire diameter of 0.5 mm had the maximum *v*/ω and ω_step-out_. The SMM’s speed–mass relationship revealed nonlinear laws that distinguish SMMs from biological systems and other artificial actuators. The optimal speed is reached at 0.0027 g with a speed of 5.42 BL/s.

### Multimodal motion, hydrodynamic disturbances, and long-distance control of SMMs

**Fig. 4.**
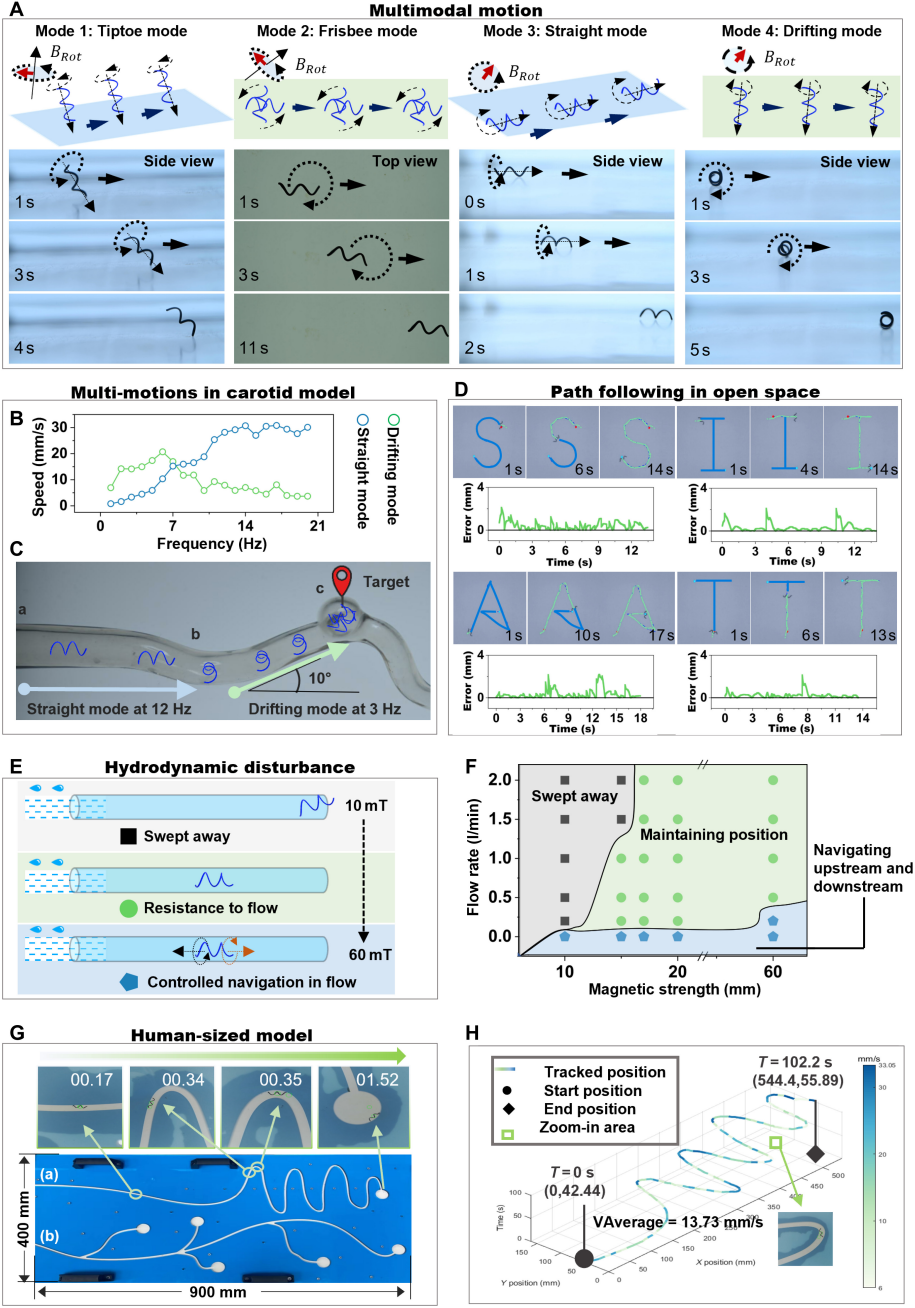
Multimodal motion adaptation of the SMM in a carotid model, hydrodynamic disturbances, and long-distance control in a human-sized model. (A) Schematic diagrams and corresponding screenshots illustrate the 4 motion modes of the SMM (Movie S2). (B) The drifting and straight modes of the SMM coexist in the same magnetic field. Below 7 Hz, the SMM with drifting mode dominants, and beyond 7 Hz, the SMM with straight mode dominants. (C) The SMM with different model motions was adapted in a carotid artery model with gradients. The horizontal sections are labeled as a–b pass by the SMM with straight mode, and the sections with a 10° slope are labeled as b–c pass by the SMM with drifting mode (Movie S3). (D) Path following of “SIAT” in open space with an average error of 310 μm (Movie S4). (E) Three states of the SMM under the impact of water flow with different magnetic fields are demonstrated, including being swept away, maintaining its position, and controllably navigating upstream and downstream. (F) The SMM shifted in the 3 states with increasing water flow and magnetic field strength. (G) A human-size model measuring 900 mm long and 400 mm wide was constructed to mimic the relative straightness of the aorta and the tortuosity of the distal vessels (Movie S5). Screenshots of target tracking in path (a) are shown simultaneously. (H) Performance metrics of the target-tracking algorithm in the human-sized model.

The SMMs show 4 motion modes within different magnetic fields, each determined by the angular inclination and frequency of the external magnetic field (Fig. 4A and Movie S2). The SMM shows tiptoe mode in a rotating magnetic field with an 80° inclination and a frequency of 15 Hz. The SMM with tiptoe model has the potential to puncture tissue for drug delivery. The SMM shows frisbee mode in a rotating magnetic field at 40° inclination and 1 Hz frequency. The SMM with the frisbee mode has the potential to facilitate drug diffusion. The SMM swims with a combination of drifting and straight modes in a rotating magnetic field with an inclination angle of 0°. Below 7 Hz, the SMM with drifting mode dominates, and greater than 7 Hz, the SMM with straight mode dominates (Fig. 4B). The straight mode of SMM is ideal motion due to magnetic torque, and the drifting mode of SMM is defined as non-ideal motion due to the wall effect [55]. The SMM with drifting mode can climb slopes of up to 22°, while the SMM with straight mode can only climb slopes of 9° with the same magnetic field strength of 6 mT (Fig. S8). This different uphill ability is related to the cost of transport, which was analyzed across the 4 modes (see Text S2 and Fig. S9). The SMM with tiptoe and straight modes consumes more energy than those with frisbee and drifting modes. The multi-motion adaptation of SMM was validated in the carotid model with gradients (Fig. 4C and Movie S3). SMM with the straight mode could travel a 0° slope (“a” to “b” in Fig. 4C) in 7 s at 12 Hz with a velocity of 1.07 BL/s. However, it was unable to climb a 10° slope in the same magnetic field of 6 mT (“b” to “c” in Fig. 4C). By reducing the frequency to 3 Hz and switching to the SMM with drifting mode, it was able to reach the target location on a 10° slope with an average speed of 0.26 BL/s. The path-following ability of the SMM with drifting mode in open space was tested with the “SIAT” path with an average error distance of 310 μm (Fig. 4D and Movie S4). The distance error is relatively small at 3.875% of the length of the robot.

Figure 4E illustrates 3 states of the SMMs when exposed to increasing magnetic field strength under the same influence of a water stream: being swept away, maintaining its position, and controllably navigating upstream and downstream. Figure 4F reveals 2 laws in this hydrodynamic disturbance. First, as water flow velocity increases, the weaker the ability of SMM to resist it in the same magnetic field. Second, the capability to resist the same water flow increases as the magnetic field strength increases. When the water flow rate is 200 ml/min, the magnetic field with 1 Hz, 60 mT can control the SMM to do controllable navigation with V_downstream =9.09 mm/s, V_upstream = 7.69 mm/s.

A model measuring 900 mm × 400 mm (Fig. 4G) and an open-space MAS were developed. The model features two 5-mm-wide laser-cut paths (Fig. 4G). Path (a) starts smooth and then becomes curved, mimicking the body’s relatively straight aorta and distal tortuous branching vascular system. Path (b) has bifurcated branches. The MAS integrates an electromagnetic array with 3 electromagnets, a UR-10 robot, and an imaging system (as shown in Fig. 1B and Fig. S10). It can generate dynamic 3-dimensional (3D) magnetic fields of 10 mT and 25 Hz within a 2.6-m spherical workspace. The SMM with θ = 63°, *r* = 0.5 mm, *L* = 8 mm, and ρn = 20% swam in path (a) under MAS with a frequency of 10 Hz and a magnetic field strength of 6 mT (Movie S5). Real-time positioning and speed metrics were captured by the imaging system of MAS (as shown in Fig. 4H). The velocity of SMM varied between 6 and 33.5 mm/s, which is 0.75 BL/s and 4.19 BL/s. The SMM completed 1.4 m of track in 102.2 s with an average speed of 13 mm/s. In brief, SMMs’ adaptability, controllability, and robustness were demonstrated through multimodal adaption in the carotid artery model with gradients, path tracking in open space, hydrodynamic disturbances, and long-distance control in the human-sized model.

### In vivo validation of the multistage embolization strategy

**Fig. 5.**
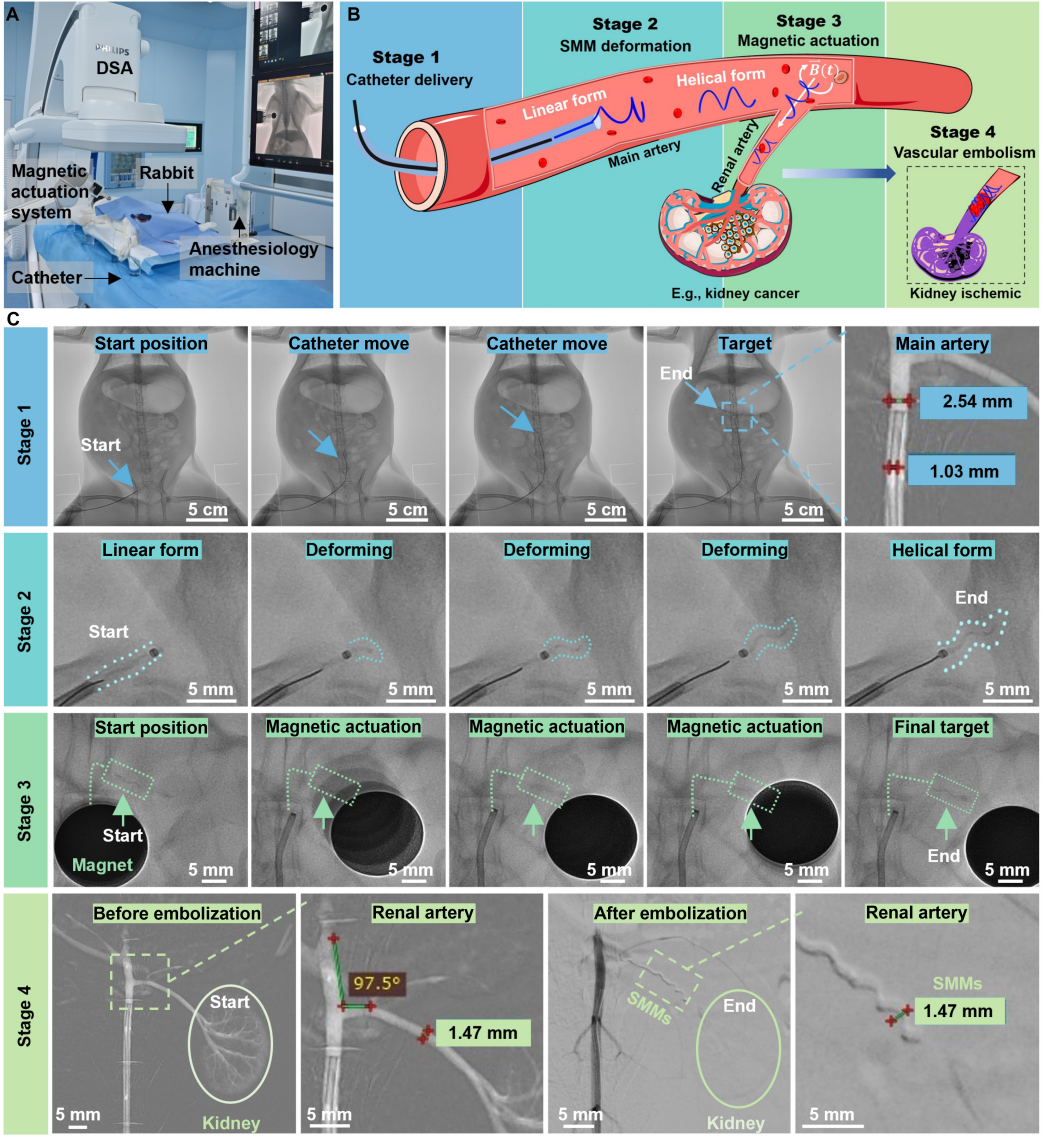
Validation of the multistage strategy for kidney embolization of the rabbit (Movie S6). (A) Experimental setup of the multistage embolization strategy in clinical application. An enlarged version of (A) is available in Fig. S11. (B) Schematic of the multistage kidney embolization strategy in a rabbit. (C) DSA chronologically captured the 4-stage embolization process inside the rabbit. Stage 1: A catheter (D_catheter = 1.02 mm) was inserted via the femoral artery to the junction between the aorta and the renal artery. The linear SMM (D_linearSMM = 0.5 mm) was then inserted into the catheter’s end and pushed rapidly by a guidewire to the catheter's head. Stage 2: The linear SMM was pushed out of the catheter into the blood and transformed into a helical shape within 2 s in blood temperature. Stage 3: Helical SMMs reached the embolization site with a rotating magnetic field. Stage 4: Angiography images before and after embolization. Before embolization, the shape of the kidney and branching vessels within the kidney were clearly visualized. After embolization, the kidney became invisible, indicating the complete renal artery embolization (D_renal = 1.47 mm, D_helicalSMM = 1.47 mm).

Here, the renal artery is taken as an example to showcase the multistage embolization capabilities (Fig. 5 and Movie S6). Figure 5A shows an integrated platform consisting of DSA, the MAS, the anesthesiology machine, a catheter, and a guidewire. A more detailed, enlarged version of Fig. 5A is available in Fig. S11. The entire procedure was visualized using DSA imaging, which offers high diagnostic quality, excellent blood flow detection, and real-time imaging (see Table S4 for comparisons with magnetic resonance imaging, B-mode, Doppler ultrasound, and endoscopy).

The renal artery connects the aorta to the kidney (as shown in Fig. 5B). When the kidney manifests malignant characteristics, e.g., cancer, and the patient is unsuitable for surgical resection, angioembolization is often the required treatment [56]. As mentioned in the Introduction section (Fig. 1), the overall strategy can be divided into 4 steps, including catheter delivery, microrobot deformation, magnetic actuation, and vascular embolization. Figure 5C shows the entire 4 stages under real-time DSA fluoroscopy, and Table shows the time consumed at each stage. In stage 1, a catheter was inserted through a puncture point in the rabbit’s right femoral artery, crosses the aorta (2.46 mm in diameter, 17.46 mm in length), and reached the junction between the aorta and the renal artery in about 1 min. The linear SMM with a 0.5-mm diameter was then inserted into the catheter’s end and pushed rapidly by a guidewire with a diameter of 0.889 mm to the catheter’s head in about 20 s. The total length of the catheter is 100 cm. In stage 2, this linear SMM was pushed out from the catheter and transformed into a preprogrammed helix within 2 s at blood temperature, with a helical diameter of 1.47 mm. Based on the diameter of the vessel and the movement efficiency of the SMM, an SMM with a diameter of 1.47 mm, a helix angle of 63° (pitch = 2.3 mm), and a total length of 4.6 mm was used in this embolization. In stage 3, the helical SMM swam into the renal artery with the actuation of a rotating magnetic field, traveling a distance of 1.52 cm within 5 s. In stage 4, the angiography clearly visualized the branching vessels within the kidney before embolization. After 3 helical SMMs reached their designated locations in the renal artery, the kidney became invisible in angiography, meaning there was no blood flow in the kidney, indicating complete renal artery embolization. As the number of SMMs increases from 0 to 3, embolization becomes more complete, with complete embolization at 3 SMMs (Fig. S12). If more than 3 SMMs are added, it may cause ischemia and necrosis in healthy surrounding tissues or organs such as the adrenal gland. Moreover, too many SMMs may result in increased pressure on the vessel walls, changes in blood pressure, increased cardiac load, etc., which may have a detrimental effect on the patient’s overall health status.

**Table.** The table shows the time consumed at each stage of this multistage embolization

	Process	Imaging mode (DSA)	Actuation mode	Distance	Time	Speed
**Stage 1**	The catheter entered through the femoral artery puncture point in the thigh and advanced to the junction of the aorta and renal artery.	X-ray imaging and angiography	Catheter moving	~17.64 cm	~1 min	2.94 mm/s
	Linear SMMs were pushed by the guidewire to reach the catheter head.		Guidewire pushing	100 cm	~20 s	50 mm/s
**Stage 2**	SMMs deformed from linear shape to helix at blood temperature.	X-ray imaging	Blood temperature	–	~2 s	–
**Stage 3**	Helical SMMs moving to target position under rotating magnetic field	X-ray imaging	Magnetic field	1.52 cm	~5 s	~3.18 mm/s
**Stage 4**	Confirmation of vascular embolization	Angiography	–	–	–	–

To conclude, complete kidney embolization was achieved by SMMS within 2 min using the multistage embolization strategy (as demonstrated in Movie S6), which covered a total transport length of over 100 cm and having a turn of 97.5°. The linear SMM inside the catheter was pushed by a guidewire at a velocity of 50 mm/s, which is 15.7 times higher than the maximum speed of the SMMs with magnetic actuation inside the body, which was 3.18 mm/s (Table). With this, the catheter was treated like a highway to linear SMM, ensuring time efficiency. Moreover, the SMMs can swim against the blood flow, as shown in the Movie S7.

### Postoperative observation

**Fig. 6.**
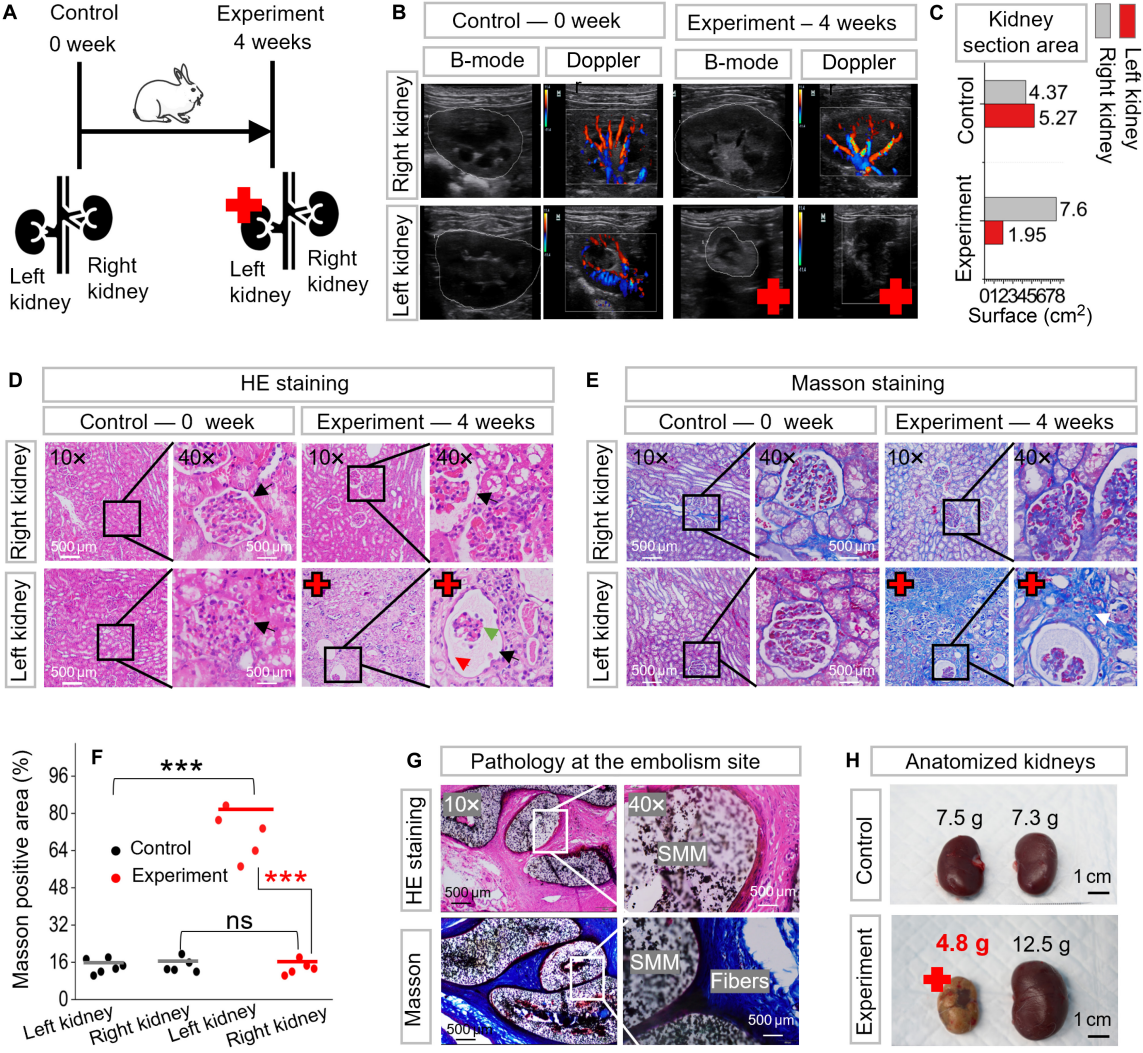
Four weeks of postoperative observation on the kidney embolization with SMMs. Red crosses indicate the embolized kidney. (A) Schematic representation of a 0-week control rabbit versus a 4-week experimental rabbit. The left kidney of the experimental rabbit had received multistage embolization for 4 weeks. (B) Observation of embolization in vivo with B-mode and Doppler ultrasound. B-mode revealed a marked reduction in the cross-sectional area of the embolized kidney compared to other kidneys. Doppler showed the blood flow status with red and blue signals, indicating a complete lack of blood flow in the embolized kidney. (C) The maximum cross-sectional area of the embolized kidney decreased from an initial 5.27 cm^2^ to 1.95 cm^2^ as measured by B-mode. (D) The renal parenchyma of the embolized kidney was severely damaged via hematoxylin and eosin (H&E) staining. (E) The extent of fibrosis in the embolized kidney was heavier than in other kidneys with Masson's staining. (F) Quantitative analysis of fibrosis as indicated by Masson's staining. The area of Masson-positive staining was significantly higher in embolized kidney than in other kidneys. “***” denotes statistical significance (*P* < 0.001); “ns” denotes no statistical significance (refer to Table S5 for details of asterisks and ns ratings). (G) Histopathological findings of the renal artery embolization site. It showed vascular wall and fibrous tissue growth tightly against SMM, confirming its biocompatibility (the optical image of the dissected renal artery is in Fig. S13). (H) Optical images of the dissected kidneys.

After the embolization, 4 weeks of postoperative observation of the rabbits was performed to evaluate the embolization ability and biocompatibility of SMMs. The left kidney of the experimental rabbits was embolized with SMMs, marked with a red cross (Fig. 6A). The renal section size and blood flow status in vivo were observed by noninvasive ultrasound imaging (as shown in Fig. 6B). The B-mode outlines the largest cross-section of the kidneys in white (Fig. 6B) demonstrating that the embolized kidney is smaller than the other kidneys. Doppler ultrasound was used to estimate real-time blood flow in the kidneys, which is an effective tool for assessing the effect of embolization, with red and blue signals corresponding to the direction of the red blood cells toward and away from the source of the ultrasound, respectively. The Doppler results in Fig. 6B showed a complete absence of blood flow signal in the embolized kidney marked by the red crosses, showing that SMMs maintain complete occlusion after 4 weeks. In contrast, the other kidneys had marked hemodynamic signals. The maximum cross-sectional area of the embolized kidney was reduced from 5.27 cm^2^ to 1.95 cm^2^ as measured by B-mode, shrinking by 62.88% as shown in Fig. 6C. The right kidney increased from 4.37 cm^2^ to 7.6 cm^2^ with an increase of 73.74% due to compensatory growth. Compensatory growth is a biological adaptive mechanism in which the body tries to compensate for the loss of an organ or tissue by increasing the size or function of another organ of the same kind.

The kidney’s pathological features were evaluated through hematoxylin and eosin (H&E) and Masson’s staining (Fig. 6D and E). The renal parenchyma of the embolized kidney was severely damaged in H&E staining (Fig. 6D). The black arrow shows the glomerular structure, the green arrow shows the capillary collaterals within the glomerulus with marked atrophy, and the red arrow shows the glomerular cystic lumen, which is relatively dilated and enlarged due to the atrophy of the capillaries. The contralateral unembolized right kidney exhibited healthy renal structures, as did the control group. Masson staining (Fig. 6E) shows the fibrotic state of the kidneys, where the area and depth of green staining represent the degree of fibrosis. The Masson staining of the embolized kidney exhibited a deeper green coloration than other kidneys. Figure 6F shows quantitative analysis of Masson staining as measured by ImageJ. Embolized kidney and other kidneys were compared as significant differences (marked as “***”, *P* < 0.001, *t* test). No significant difference in kidney health between contralateral unembolized kidney and controls (marked as “ns”, *P* = 0.05, *t* test). More details about the *t* test are available in Table S5.

Figure 6G shows a histopathological investigation of the embolized site, where the cell wall and collagen fibers grew tightly against the SMMs, demonstrating high biocompatibility. The dissected optical picture of the embolization site is shown in Fig. S13. Observing the dissected kidneys (Fig. 6H), the embolized kidney weighed only 4.8 g, representing a 36% reduction compared to the 7.5-g left kidney of the control group. In contrast, the right kidney showed a 71.2% increase in size from 7.4 g to 12.5 g due to compensatory growth. The histocompatibility of the SMM was investigated by implanting a 20-mm straight SMM into a rabbit’s back for 1 week (Fig. S14). From the optical image and H&E staining results shown in Fig. S14, the SMM is tightly encapsulated by the surrounding tissue 1 week after implantation, which indicated good immunological responses on surrounding tissues [57,58].

To conclude, 4 weeks after the operation, no blood flow signals in the embolized kidney were observed by Doppler ultrasound, demonstrating that the SMMs could maintain their embolic position for an extended period. Marked kidney atrophy was observed by B-mode and dissected kidney. The high biocompatibility of the SMMs was demonstrated by H&E and Masson staining, with the contralateral kidney remaining healthy and the surrounding tissue growing tightly against the SMMs. Our multistage strategy’s non-migration, maintenance of complete embolization, and biocompatibility were demonstrated in vivo by long-term postoperative follow-up.

## Discussion

Current interventional embolization is performed by delivering the material directly to the target site through an interventional catheter. Embolic materials include coils, stents, microspheres, gelatin sponge particles, polymers, protein sponges, hydrogel microspheres, and liquid formulations, but their practical performance and long-term results remain unsatisfactory. The reasons for this include difficulty in reaching tortuous distal vessels, the possibility of incomplete embolization, and leakage and fragmentation of the embolic material leading to distal occlusion of the accidental artery [31]. In this study, the multistage embolization strategy addresses the difficulty of reaching distal vessels, the challenge of complete embolization and prolonged stability of the embolus, and the validation of biocompatibility. The novel multistage vascular embolization strategy is developed using the catheter-assisted tetherless swimming SMM for vascular embolization in hard-to-reach regions. It provides new solutions for vascular diseases such as aneurysms, cerebral aneurysms, tumors, hemorrhages, varicose veins, acute pulmonary embolism, and renal disease (please refer to Table S1 for current limitations and our contribution for these diseases). As demonstrated in the Introduction and In vivo validation of the multistage embolization strategy sections, this catheter-assisted SMM multistage embolization strategy consists of 4 stages: catheter delivery, SMM deformation, magnetic actuation, and embolization. The speed of linear SMM inside the catheter is 50 mm/s, which is 15.7 times higher than the maximum speed when the SMM is under magnetic actuation inside the body, which is 3.18 mm/s. With this, the catheter is treated like a highway to linear SMM, ensuring time efficiency. A catheter with a 1.02-mm inner diameter transported an SMM with a 1.47-mm diameter, demonstrating that catheter-assisted deformable microrobots do not require catheter enlargement as needed for fixed-size tool transport. Furthermore, using the SMM’s wireless magnetic actuation to reach hard-to-reach vessels compensates for the catheter’s shortcomings in tortuous vessels. The catheter and deformable SMMs mutually benefit each other.

SMMs, made of specialized organo-gel and NdFeB, can be easily fabricated due to the suitable shear viscosity (608 to 982 mPa.s) at room temperature and can be formed in just 30 s under UV light. We obtain the optimal parameterized SMM with a 20% magnetic particle concentration, a 63° pitch angle, and a 0.5-mm wire diameter. The optimal SMM shows excellent biocompatibility in pathology and high radiographic visibility in x-ray imaging. They also demonstrate outstanding shape memory properties, with 92.46% shape fixation and 82.79% shape recovery. Table S2 demonstrates the properties of SMM compared to other shape memory materials, highlighting SMM’s shape memory performance. Additionally, the SMM shows rapid dual responses to thermal stimulation and magnetic fields. They can transform from a linear SMM into a predetermined helical SMM within 2 s in blood temperature. The helical SMM weighing 0.0027 g can achieve a maximum speed of 5.08 BL/s under a weak magnetic field of 6 mT, exhibiting a superior body mass–speed ratio (Table S3). They can efficiently navigate through tortuous vessels and against the blood flow by mimicking the motion of bacterial flagella.

Current challenges in the field of microrobots include adapting to complex environments, resisting disturbances, developing magnetic actuation platforms with larger workspaces, and enhancing in vivo tracking applications. Therefore, we investigated the adaptability, robustness, and controllability of SMMs in various scenarios. We demonstrated the adaptability of SMM to 3D carotid models with gradients by switching motion modes. We achieved path tracking of the SMM with an average error of only 3.875% BL in open space. Additionally, the hydrodynamic testing of the SMM in water flow showed that it could withstand a stream of 200 ml/min at 1 Hz and 60 mT, maintaining upstream and downstream velocities of 0.96 BL/s and 1.14 BL/s, respectively. A human-sized model and MAS for generating dynamic 3D magnetic fields within a 2.6-m-diameter spherical workspace have been developed, allowing the realization of long-distance magnetic actuation of SMMs. Furthermore, an integrated platform combining DSA, MAS, a catheter, and a guidewire has been developed to ensure rapid, accurate, real-time deployment and actuation of SMMs in vivo.

Currently, most wirelessly actuated microrobots are primarily tested in vitro and lack further exploration in clinical trials. In this study, we validate the whole multistage procedure in live rabbits, achieving controlled wireless actuation with and against blood flow. The SMM completes a remarkable 100-cm travel and renal artery embolization in 2 min, all under real-time fluoroscopy. Four weeks after the surgery, the SMMs can maintain complete vascular embolization in vivo, as confirmed by Doppler showing no blood flow in the embolized kidney. SMMs show high biocompatibility, as confirmed by H&E and Masson staining, showing that the contralateral kidney remains healthy and the vessel wall grows tightly against the SMMs. The size of the embolized kidney is reduced by 36%. Long-term postoperative follow-up demonstrated the non-migration, maintenance of complete embolization, and biocompatibility of the proposed multistage strategy. Our strategy integrates shape memory materials and wireless magnetically driven microrobots into modern medical devices to ensure efficient and practical embolization in hard-to-reach vascular regions. We also encountered several challenges in animal experiments. Firstly, the workspace of a 3D Helmholtz coil, as we use for in vitro experiments or small animals, such as rat experiments, is unsuitable for open human-sized space in clinical settings. In this study, an electromagnetic array carried by a robotic arm was developed as a magnetic actuation, which can generate a magnetic field in a spherical space with a diameter of 2.6 m. Secondly, the magnetic field strength is not strong enough, and the magnetic field decays too fast to generate sufficient field strength in the target area. In the rabbit experiments of this study, the problem of magnetic strength was addressed in 2 ways: One method is to optimize the design of the robot by optimizing the material formulation, e.g., magnetic particle content, and optimizing the geometry, e.g., helix angle, to obtain the best energy transfer efficiency of the robot. Another method is replacing the electromagnetic array (with a magnetic field strength of 10 mT) with a rotating permanent magnet (with a stronger magnetic field strength measuring 756 mT). The permanent magnet generates enough field strength in the target area to control the robot’s precise movements, even when separated from biological tissues by a few centimeters.

Looking ahead, we aim to extend our experiments to various models, including malformed vascular embolization, malformed arterial aneurysms, and cerebral aneurysms, thereby enhancing the generalizability of our findings. For these diseases, our SMMs may have difficulties as follows. Firstly, the standard helical-shaped SMMs may not fully meet the embolization needs for aneurysmal and vascular malformations. To address this issue, it is necessary to create SMMs with different shapes tailored for the corresponding malformation vessel. In addition, hand-made SMMs are dimensionally unable to meet the requirements for the embolization of capillary-like scale vessels. To address this issue, new technologies such as fine 3D printing, photolithography, atomic layer deposition, and electron and ion beam processing can be utilized.

## Materials and Methods

### Materials

DDFD (CAS: 13031-04-4, Bidepharm) was used as photoinitiator; ACMO (CAS: 5117-12-4, Macklin), BMA (CAS: 2495-37-6, Macklin), 2-EHA (CAS: 103-11-7, Macklin), and tBA (CAS: 1663-39-4, Macklin) were used as monomers; TAPUA-B368 (CAS: 68987-79-1, Wuhan Huaxiang Kejie Biotechnology Co., Ltd) was used as the crosslinker; NdFeB (CAS: 918106-59-9, Guangzhou Zhuoli Machinery Factory) was used as the magnetic particle.

### Fabrication of the SMM

The organo-gel precursor solution contained 25 wt% ACMO, 12.5 wt% BMA, 7.5 wt% 2-EHA, 4 wt% tBA, 1 wt% DDFD, and 50 wt% TAPUA. They were well mixed in a magnetic stirrer for several minutes at room temperature, then mixed with different ratios of magnetic particle NdFeB (5 to 50 wt%).

A syringe (1 ml capacity) and a needle (stainless steel, 26G, 0.45 mm outer diameter [OD], 0.24 mm inner diameter [ID]; 30G, 0.31 mm OD, 0.16 mm ID) attached were used for injection. The solution was injected into a transparent silicone tube (0.8 mm OD, 0.5 mm ID; 0.8 mm OD, 0.3 mm ID). The solution-filled silicone tube was twisted on a threaded mold. The drawing of the mold was designed with the software SolidWorks 2021 and printed in WENECT Ltd. The geometry of the mold determines the diameter and the pitch of the helix. After winding on the mold, the tube was exposed to UV light (light source: Lin Shang UV, 50 W, 405 nm, 1,200 to 2,000 mW/cm^2^ under 2 cm distance) for 30 s at room temperature, and then placed into a magnetizer (electromagnet, WD-60) with a 750-mT magnetic field perpendicular to the helix axis. Finally, straighten the tube and pull out the linear SMM inside at 20 °C. The linear SMM can be quickly transformed into a predetermined helix at temperatures above 38 °C.

### Methods of velocity measurement during the optimization of SMM

In this paper, different magnetic particle concentrations, different geometries, and their motions under different magnetic field strengths and solution viscosities were tested. A glass bottle with a straight 5-mm-wide channel was placed inside a 3D Helmholtz coil to measure the speed of SMMs. Utilize vision-based pixel target recognition and frame difference velocimetry to locate and measure the speed of the SMM in real time. For each test, 60 velocities around the middle of the channel were taken to calculate the average velocity and error after removing the extreme values. For the speed test in solutions of different viscosities, the temperature of each solution was controlled at 38 °C.

### In vivo embolization

The Animal Ethical Committee of Shenzhen Institute of Advanced Technology, Chinese Academy of Sciences, China: SIAT-IACUC-220714-JCS-XTT-A2165 approved the animal experiments. The laboratory animals’ care followed institutional guidelines of the Chinese Academy of Sciences and the Council for the Purpose of Control and Supervision of Experiments on Animals, Ministry of Public Health, China.

Two New Zealand white rabbits weighing 3 kg were used for in vivo embolization. The rabbit was bought from the Guangzhou Suibei Breeding Center (Guangzhou, China). The rabbits were kept under standard feeding conditions, with access to sufficient water, an appropriate amount of feed, and a suitable temperature. Food was withheld for 6 h prior to surgery. Before the operation, SMMs, with diameters varying from 0.8 to 2 mm, were pre-fabricated in 0.1-mm increments to match potential renal artery inner diameters. Before surgery, the rabbit was anesthetized using isoflurane via an RWD gas filter canister for 10 min and subsequently secured to an acrylic anatomical platform. After shaving the base of the thigh fur and sterilizing the exposed skin, a 4F sheath was inserted through the surgically exposed femoral artery. This procedure was continuously monitored using a ZEISS TIVATO700 stereomicroscope. The rabbit was then positioned on an operating table equipped with DSA (Philips, Netherlands) and magnetic control instruments. A 4F catheter (1.02 mm internal diameter, 100 cm length), accompanied by a guidewire (0.889 mm diameter, 150 cm length), was inserted for selective renal angiography. Clear visualization of the kidney and its vascular branches is shown in Fig. 5C before embolization. A multistage embolization strategy was then performed. The MAS included a UR-10 robotic arm and a spherical permanent magnet with a surface force of 756 mT and a diameter of 2 cm made of NdFeB N52. After embolization, the guidewire and catheter were removed, and the wound was then sutured. The rabbits were returned to the animal care room and injected with penicillin (penicillin sodium for injection, C_16_H_17_N_2_NaO_4_S) over 3 days to reduce the risk of infection. Three rabbits were embolized in the research, and all 3 rabbits showed marked renal atrophy under ultrasonographic examination after 4 weeks. One of them was dissected and examined by pathologic section, as shown in Fig. 6D, E, and G. The control group was a live rabbit of the same weight that had not been subjected to any experiments. Statistical analyses of the experimental and control groups are shown in Fig. 6C, F and H, all demonstrating renal atrophy.

### Postoperative assessment: Ultrasound and histological evaluation

Four weeks after surgery, the rabbit was first anesthetized in the same way as before. Then, the location of the kidneys was gently felt with a hand, and the corresponding hair was removed with a razor. An appropriate amount of ultrasound gel was applied to the shaved area, and then the size of the kidneys and blood flow were observed using B-mode and Doppler ultrasound (ultrasound brand: mindray, model: M55). Dissected kidneys, including embolized kidneys, contralateral kidneys, and embolized sites, were removed from control and experimental rabbits. Their thin tissue sections were prepared and examined histologically by H&E and Masson staining. Finally, the kidneys were preserved in paraformaldehyde fixative (4% paraformaldehyde, PB buffer) after being examined macroscopically.

### Equipment utilized

The SEM images in Fig. 2G were taken by a scanning electron microscope (Hitachi SU8000, Japan), and the rheological analysis in Fig. 2D was completed using the Anton Paar MCR302 rheometer (Anton Paar, Austria). The curing mechanism of 20% to 50% magnetic particle concentration was analyzed using FTIR acquired with a Thermofisher-Nicolet is50 (Thermo Fisher Scientific, USA) (Fig. 2). The shape memory properties (Fig. 2) of the organo-gel was quantified through DMA/thermochemical analysis (TA-Q800 machine; TA Instrument, USA), focusing on 2 primary metrics: shape recovery (Rr) and shape fixity (Rf) ratios. The selected B368-organohydrogel’s Rf and Rr were ascertained using DMA cyclic tests in the 38 °C to 100 °C temperature range, offering insights into the material’s shape memory characteristics. The magnetic field strength was measured with the Tesla tester (YHT03, No. 2705, YuanHenTong Technology Co).

### Data collection and analysis software

DSA 3D workstation: interventional tools 1.4.6; DSA software kit: Philips 7M20 1.1.5; Stereomicroscope software kit: CARL ZEISS TIVATO 700 1.6.0.741; Mechanical software kit: depex 4.0.1; DMA software kit: TA Instruments Universal Analysis 2000 4.5.0.5; SEM: Hitachi SU8200 3.5.3.0; Rheology software kit: NETZSCH Kinexus Lab+ 2.0.0.0; Mechanical software kit: depex 4.0.1; Masson-stained positive area: ImageJ; FTIR spectrum: Omnic 8.2.0.387; Origin 2021; RadiAnt DICOM Viewer 2020.2.3 (DSA); H&E staining and Masson staining: Maxim Medical Diagnosis MXD.

## Supplementary Materials

Supplementary 1 Texts S1 and S2 Figs. S1 to S14 Tables S1 to S5 Movies S1 to S7
