# Supplementary material for: Thermal and Magnetic Dual-Responsive Catheter-Assisted Shape Memory Microrobots for Multistage Vascular Embolization
Source: Research (Wash D C). 2024 Mar 26;7:0339. doi: 10.34133/research.0339 (PMC10976590; doi:10.34133/research.0339)
Supplement: Supplementary 1 — Texts S1 and S2 Figs. S1 to S14 Tables S1 to S5 Movies S1 to S7 [file research.0339.f1.zip › Supplementary Materials Revisions -clean version.pdf]

Supplementary Materials for

“Thermal and Magnetic Dual-Responsive Catheter-assisted Shape Memory Microrobots for Multistage Vascular Embolization”

Qianbi Peng et al.

*Corresponding author: Tiantian Xu, tt.xu@siat.ac.cn

The PDF file includes:

Supplementary Texts S1 to S2
Figs. S1 to S14
Tables S1 to S5
Legends for movies S1 to S7

Other Supplementary Material for this manuscript includes the following:

Movies S1 to S7

Supplementary Texts

Text S1

Propulsive Forces on SMMs:

Based on the kinematic analysis of micro-helical robots, the propulsion of SMMs involves both magnetic force and torque when subjected to an external uniform magnetic field with a magnetic flux intensity B. For a SMM characterized by a magnetic body volume V and volume magnetization M, the magnetic force and torque can be expressed as:

$$\mathrm{F}_m = V(M \cdot \nabla)B$$
$$T_m = VM \times B$$

Given that only a uniform rotating magnetic field is employed for the actuation of helical micro-robots, the external magnetic force nullifies, leading to the equation for maximal magnetic torque as:

$$\mathrm{T_m} = \int_{\mathrm{V_m}} \mathrm{M} \times \mathrm{B}\, dV_m$$

$$\mathrm{T_m^{max}} = MVB = MB\kappa\pi r^2 n\sqrt{((2\pi R)^2 + \lambda^2)}$$

Here, k is the concentration coefficient, r is the wire radius, n is the number of helical turns, and R is the helical outer radius.

Drag Forces on SMMs:

The asymmetric propulsion matrix encapsulates the axial swimming of a helical propeller (Purcell 1977, 1997), which correlates four principal scalar quantities: forward velocity v, angular velocity ω, torque τ, and force f. This relationship can be mathematically represented as:

$$\begin{bmatrix} \mathrm{f} \\ \tau \end{bmatrix} = \begin{bmatrix} \mathrm{a}\ b \\ \mathrm{b}\ c \end{bmatrix} \begin{bmatrix} \upsilon \\ \omega \end{bmatrix}$$

The matrix parameters are computed as follows:

$$a = 2\pi nR(\frac{\zeta_{\|}\cos^2\theta + \zeta_{\perp}\sin^2\theta}{\sin\theta})$$
$$b = 2\pi nR^2(\zeta_{\|} - \zeta_{\perp})\cos\theta$$
$$c = 2\pi nR^3(\frac{\zeta_{\perp}\cos^2\theta + \zeta_{\|}\sin^2\theta}{\sin\theta})$$

Here, n is the number of helical turns, R is the helical outer diameter, and $\xi\perp$ and $\xi\|$are tangential and normal resistance coefficients.

$$\zeta_{\perp} = \frac{4\pi\eta}{\ln(0.36\pi R / r\sin\theta) + 0.5}$$
$$\zeta_{\parallel} = \frac{2\pi\eta}{\ln(0.36\pi R / r\sin\theta)}$$

η is the constant dynamic viscosity, r is the radius of the helix, and θ is the helical angle $\theta = \arctan(2\pi R / \lambda)$. The helical angle is calculated based on the outer diameter. The ratio v/ω signifies the sensitivity of the SMM to magnetic fields and $\omega_{step-out}$ influences the maximum velocity (Vmax) attainable by the SMM.

$$\upsilon / \omega = -\mathrm{b} / a$$
$$\omega_{step-out} = a / (ac - b^2) T_m^{\max}$$

Text S2

To determine the Cost of Transport (CoT), we proceed through a series of calculations in the following order:

First, we evaluate the total volume of the helix, Vtotal .

$$Vtotal = \pi r2 \times L \quad (1)$$

Next, calculate the volume of the magnetic particles, V, which allows us to determine the magnetic moment, m.

$$V = Vtotal \times \rho n \quad (2)$$

$$m = V \times Msat \quad (3)$$

Using this information, we compute the magnetic torque, Tm , for each mode.

$$Tm = m \times B \times \sin(\alpha) \quad (4)$$

Where B=0.006T, α is the angle between the magnetic field and the helix axis.

Subsequently, calculate the input energy, E_{input}.

$$E_{input} = Tm \times 2\pi \times f \times t \quad (5)$$

Where f is the frequency of the external magnetic field, and t is the given time. This type of energy input calculation is commonly used for periodic drive systems, and this equation assumes that the system is running at a constant frequency f for a given time t and that the magnetic moment Tm is constant.

Finally, determine the Cost of Transport (CoT).

$$CoT = \frac{E_{input}}{d * m} \quad (6)$$

Where d is the distance covered in the direction of propulsion during the given time, and m is the mass of the helical robot.

Some fundamental constants of SMM:

Msat=0.966T (Saturation magnetization)

r=0.00025m (Line radius)

L=0.008m (Total length)

m=0.0025g (Mass of our robot)

ρn = 0.2 (Magnetic particle content)

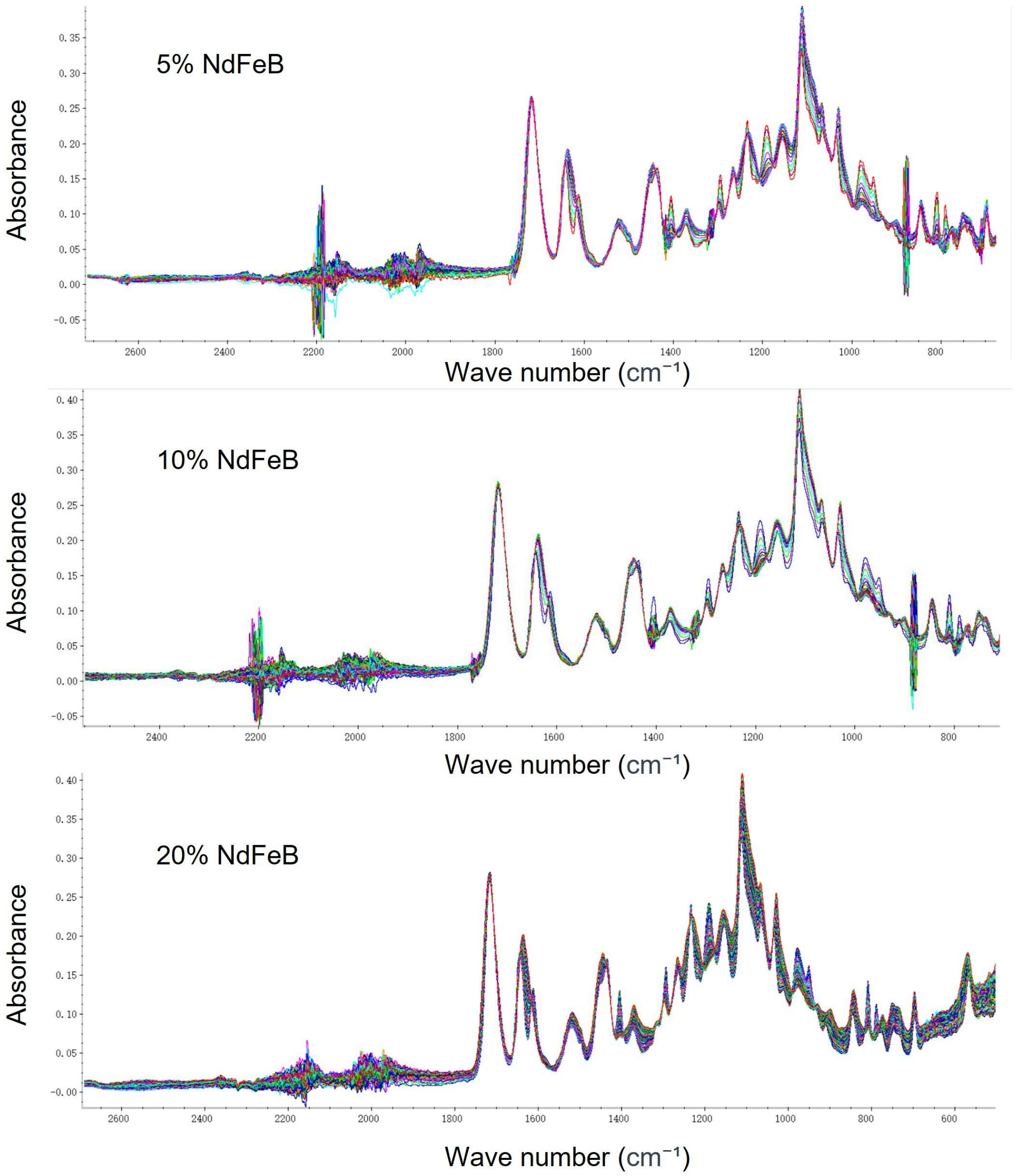

Fig.S1. Infrared Spectroscopy Chart for the solution with 5%-20% magnetic particle concentration.

5wt%NdFeB: measuring peak: 980, reference peak: 1717, curing rate=35.3%;

10wt%NdFeB: measuring peak: 980, reference peak: 1717, curing rate=29.4%;

20wt%NdFeB: measuring peak: 979, reference peak: 1716, curing rate=27.8%.

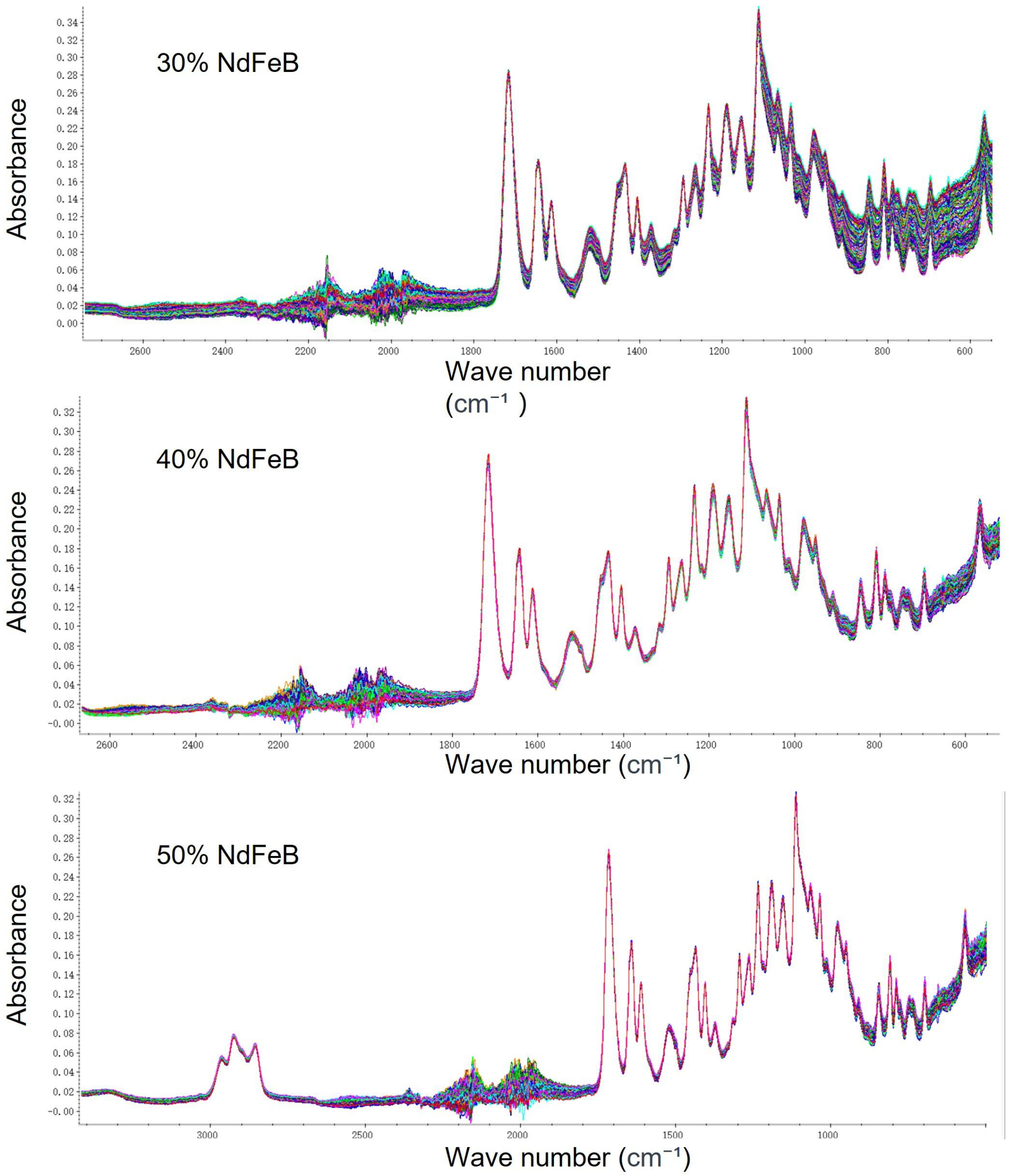

Fig.S2. Infrared Spectroscopy Chart for the solution with 30%-50% magnetic particle concentration.

30wt%NdFeB: measuring peak: 980, reference peak: 1718, curing rate=10.5%;

40wt%NdFeB: measuring peak: 980, reference peak: 1718, curing rate=5%;

50wt%NdFeB: measuring peak: 980, reference peak: 1718, curing rate=1.6%.

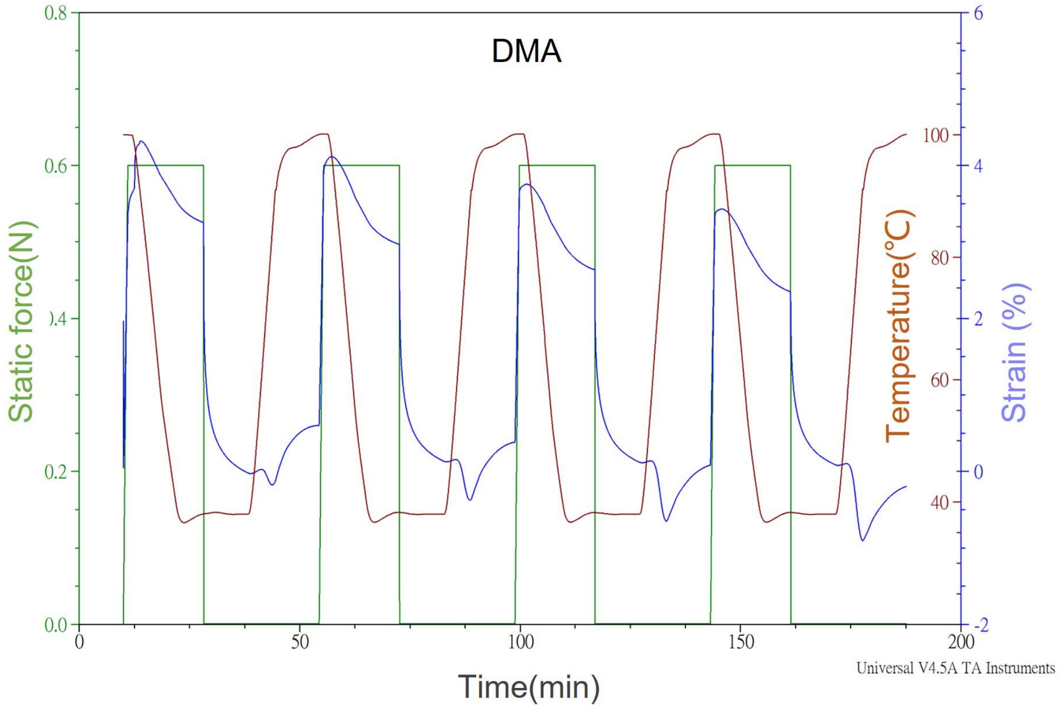

Fig.S3. DMA (Dynamic Mechanical Analysis) plot of the SMM with 20% NdFeB. The SMM exhibits a gradual decrease in 4D deformation and shape recovery rates during four DMA test cycles at 38℃ and 100℃. Rf: shape fixity ratio Rr: shape recovery ratio.

38℃:

Rf (2)=92.46%
Rf (3)=82.31%
Rf (4)=73.47%
Rr (2)=82.79%
Rr (3)=76.78%
Rr (4)=70.53%

100℃:

Rf (2)=94.68%
Rf (3)=86.92%
Rf (4)=79.17%
Rr (2)=99.82%
Rr (3)=93.34%
Rr (4)=85.42%

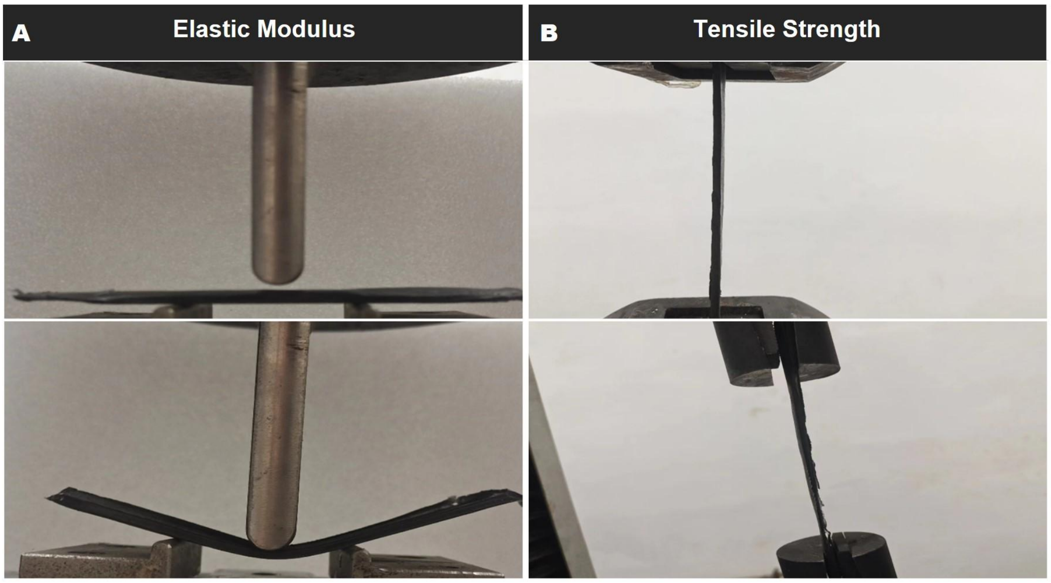

Fig.S4. Testing of elastic modulus and tensile strength of the Shape Memory Magnetically Responsive Gels. The test was carried out at 38 °C with a 5 mm/min test speed. The test material has a thickness of 1.54mm, a width of 9.99mm, a length of 90.31mm, and a magnetic particle content of 20%. **(A)** Testing the elasticity modulus of the material. **(B)** Testing the tensile strength of the material.

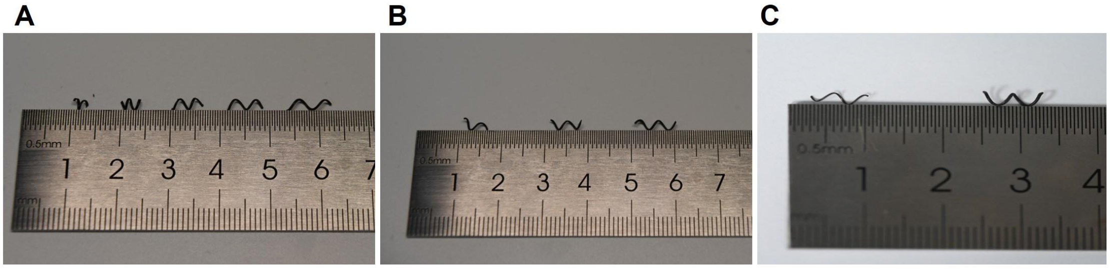

Fig.S5. The optical pictures of different geometric parameters of SMMs. (A) Pitch λ ranges from 1, 2, 3, 4 to 5mm, i.e., pitch angle θ ranges from 82.71, 75.71, 69.09, 63.01 to 57.52°. The other geometrical details remain the same, i.e., helix radius R=2.5mm, wire radius r=0.25mm, total length L=2*pitch. **(B)** Total length L from 1.5, 2 to 2.5 times pitch. The other geometrical dimensions remain R=2.5mm, r=0.25mm, λ=4mm. **(C)** The wire diameters are 0.3mm and 0.5mm. Other geometrical dimensions remain unchanged, i.e., L=2*pitch, λ=4mm.

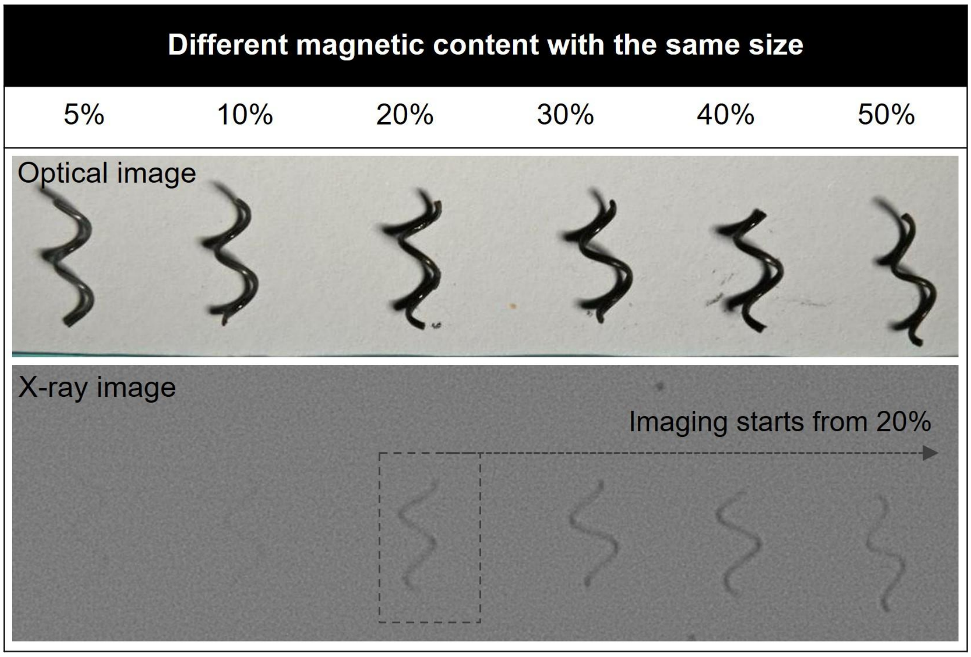

Fig.S6. Optical and X-ray images of SMMs with different magnetic particle weight ratios. In the X-ray image, the SMM with 5wt% and 10wt% magnetic particle content are almost invisible. In contrast, the SMM with 20wt% and higher magnetic particle content are clearly recognizable.

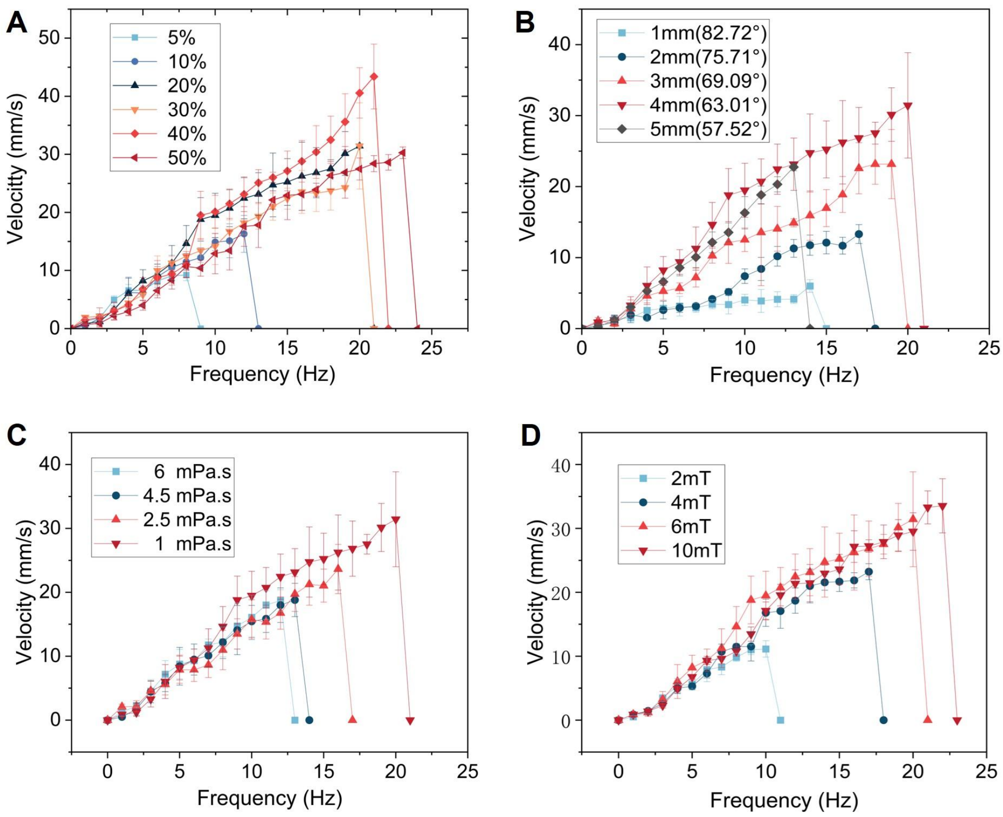

Fig.S7. The original data of speed-frequency relationship for SMMs. (A) The relationship between speed and frequency shows similar slopes (v/w) for SMMs with different magnetic particle percentages. However, the SMM containing 20-50% particles exhibits a plateau in the step-out frequency ($\omega_{step\text{-}out}$). **(B)** The speed-frequency relationships of SMMs with different pitch lengths. As the pitch increases (helix angle decreases), both the v/w ratios and $\omega_{step\text{-}out}$ first increase and then decrease, peaking at pitch λ= 4 mm (θ=63.01°). **(C)** The speed-frequency relationships of SMMs in different solution viscosities. The v/w are similar in solutions of varying viscosities, but the $\omega_{step\text{-}out}$ decreases with increasing viscosity.**(D)** The speed-frequency relationships of SMM in different magnetic field strengths. The v/w are similar for different magnetic field strengths, but the $\omega_{step\text{-}out}$ varies greatly, with a large gap between 2mT and 4mT and a significant plateau between 4mT and 10mT.

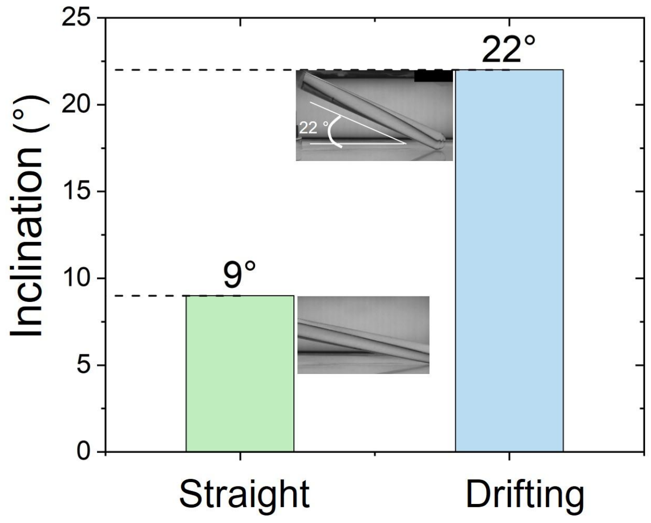

Fig.S8. Comparison of the uphill capability of the SMM with two modes in the lumen at the same magnetic field strength. The SMM with drifting mode can climb a slope of 22°, while the SMM with straight mode can only climb up to a maximum slope of 9°.

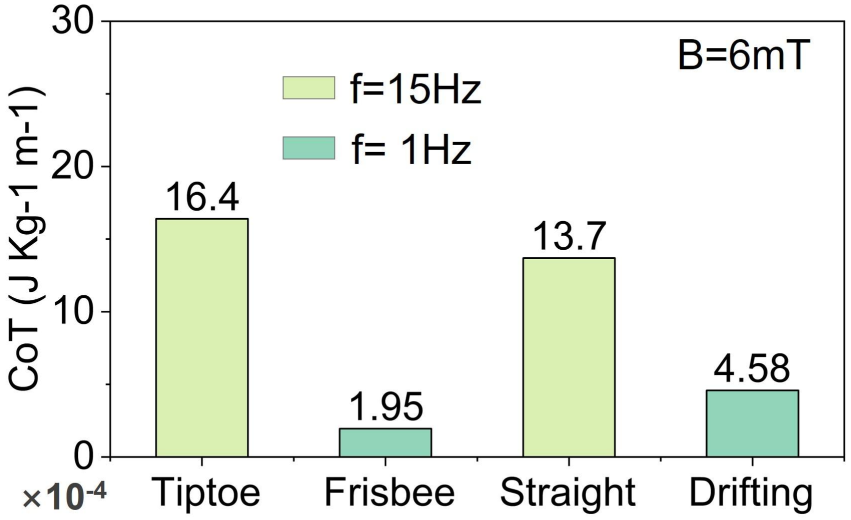

Fig.S9. Cost of transport (CoT) for the four motion modes of the SMM. A larger CoT means less efficient transmission. The graph shows that the SMM with frisbee mode has the lowest Cost of transport, which represents the most efficient. The SMM with tiptoe mode has the highest CoT for the least efficient transmission. Overall, the SMM with modes that require high-frequency actuation have lower transmission efficiencies than low-frequency ones. There is often a biological tissue spacing between the magnetic control device and the actual blood vessel in the open space, which can lead to substantial magnetic attenuation. Increasing the magnetic field strength indefinitely is challenging and increases the procedure's risk, so it makes clinical interest to look for more efficient movement patterns.

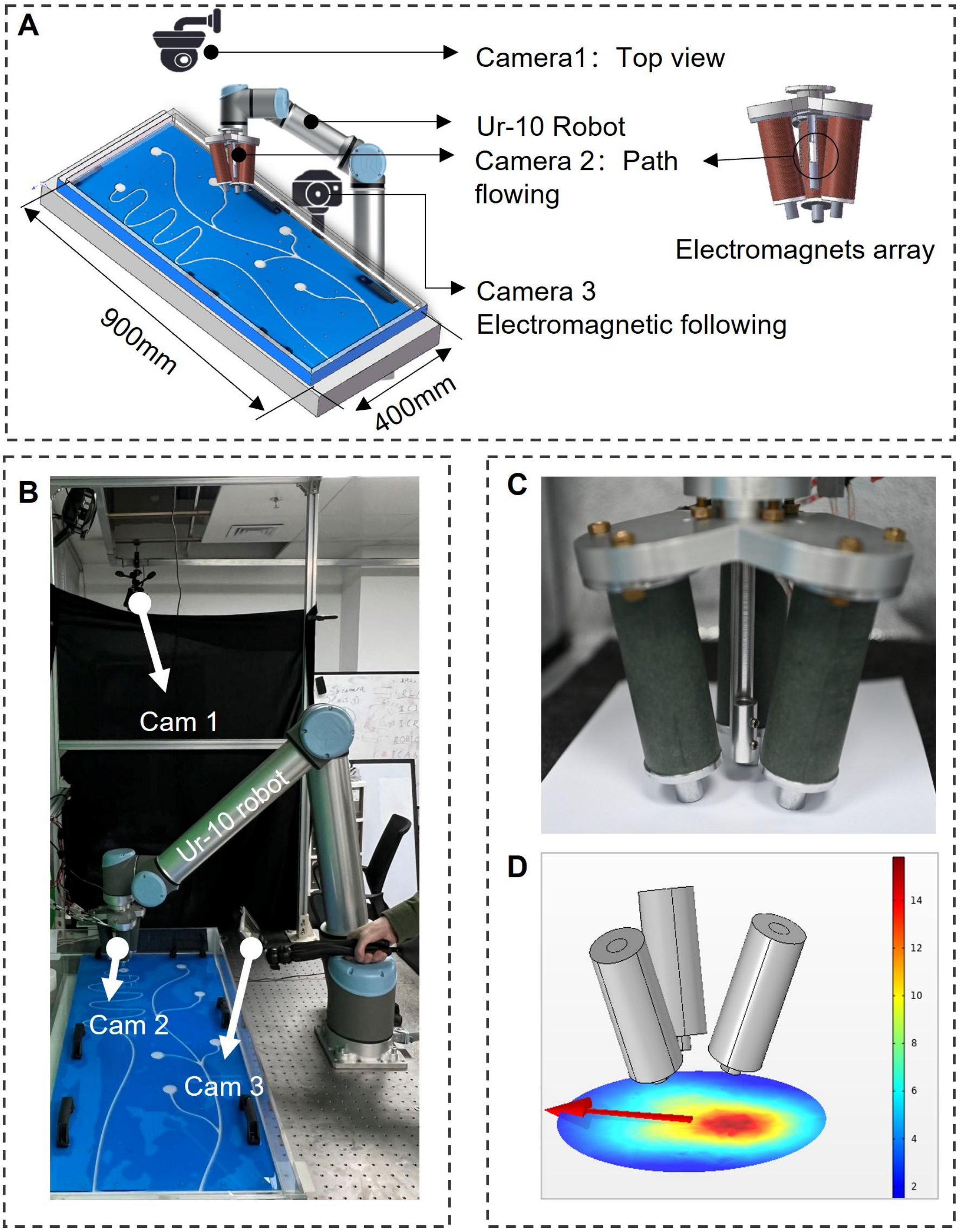

Fig.S10. **Presentation of our magnetic actuation system (MAS). The MAS is capable of generating dynamic 3D magnetic fields within a 2.6m-diameter spherical workspace. (A)** Schematic of the MAS. **(B)** Optical image of the MAS. The MAS integrates the electromagnets array with three electromagnets, a UR-10 robot, and an imaging system including 3 cameras. Camera2 is embedded with the target following algorithms, which is mounted in the middle of the electromagnets array to track the position of the SMM in real-time and calculate the velocity (movie S5). **(C)** Optical image of the electromagnetic coils array. **(D)** Schematic of the magnetic field that MAS produces. It can generate a magnetic field of up to 10 mT and 25 Hz at a distance of 5 cm.

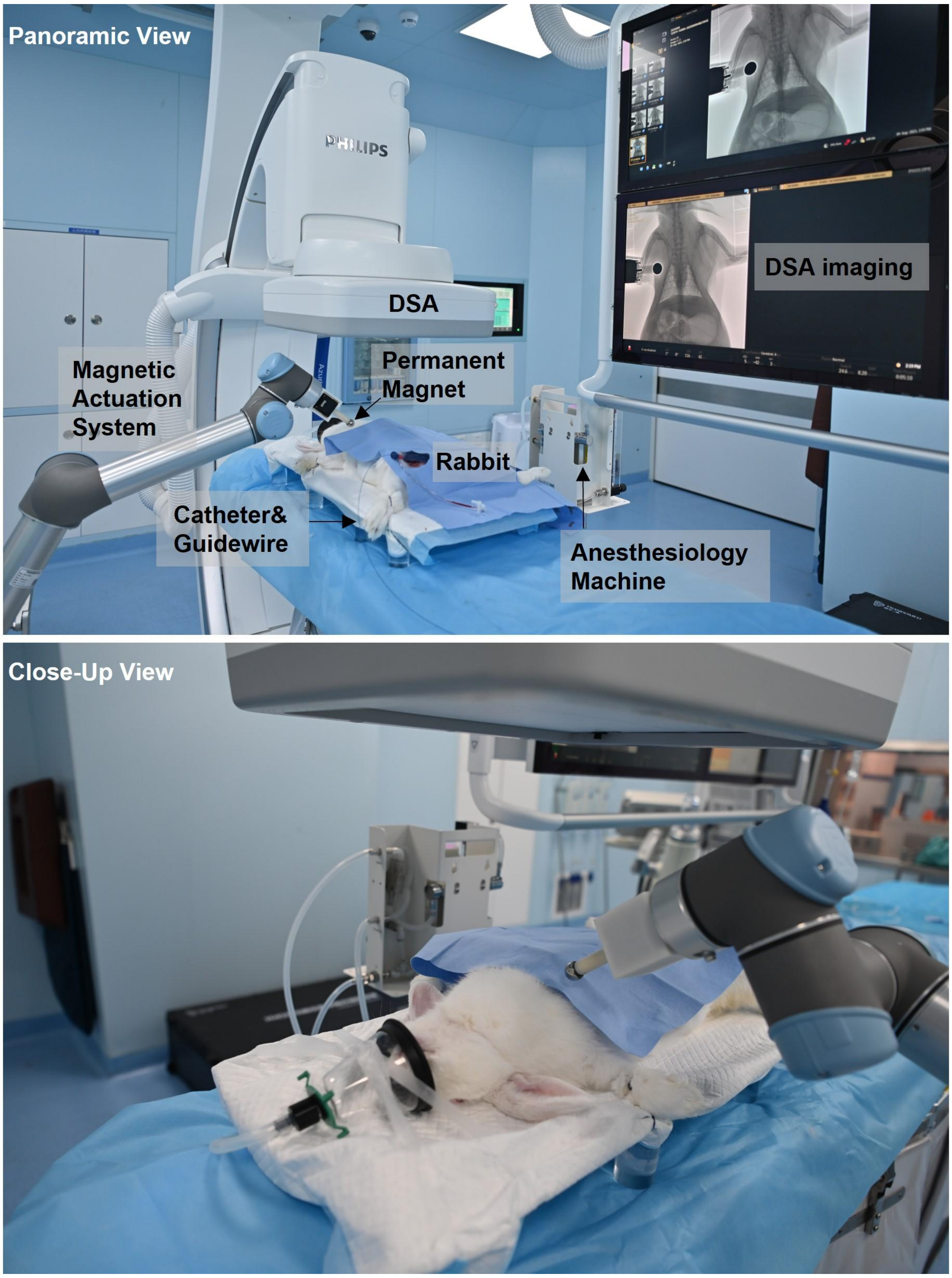

Fig.S11. Panoramic and close-up view of the rabbit experimental setup. The clinical trial includes a DAS, a screen of the DSA imaging, a MAS with a rotating permanent magnet, an anesthesiology machine, a rabbit, a catheter, and a guidewire. The spherical permanent magnet has a surface magnetic strength of 756 mT and a diameter of 2 cm.

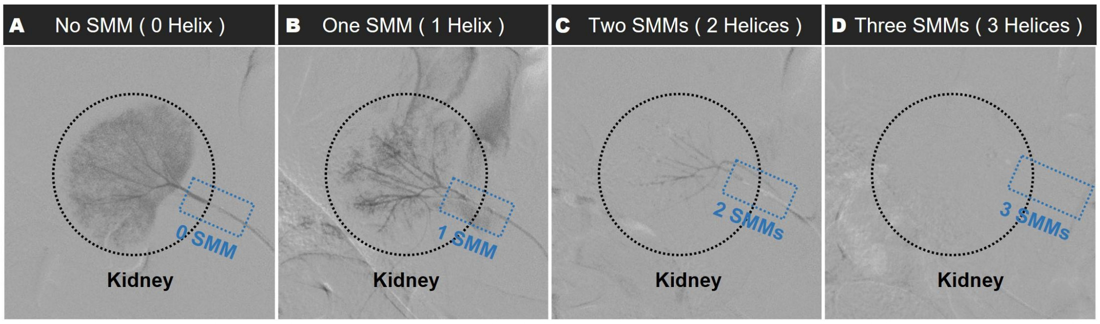

Fig.S12. Angiographic observation of the effect of different numbers of SMM on embolization in vivo. **(A)** A clear shape and the internal vessels of the kidney are visible when there is no SMM present in the renal artery. **(B)** Some of the internal vessels of the kidney are visible with one SMM in the renal artery. **(C)** Very few internal vessels of the kidney are visible with 2 SMMs in the renal artery. **(D)** The kidney is not visible with 3 SMMs in the renal artery.

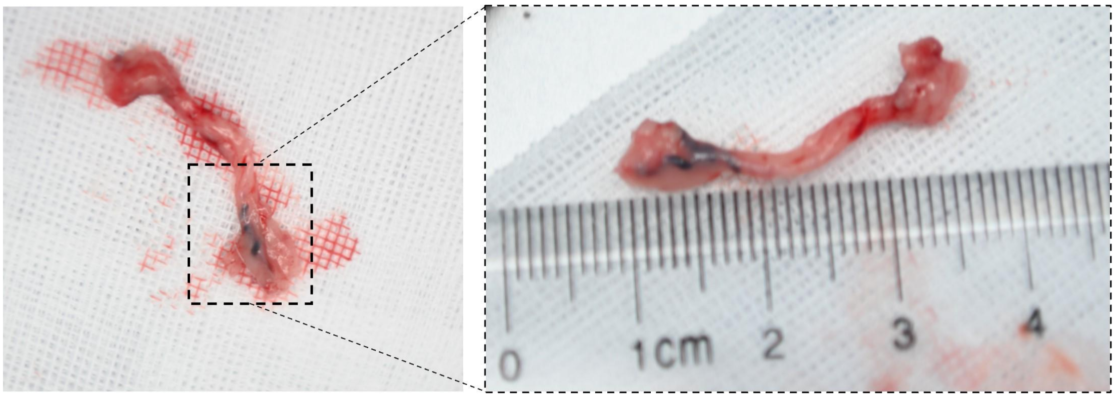

Fig.S13. The anatomical picture displays the renal arteries of the embolized kidney at four weeks postoperatively. SMMs are clearly visible, intertwined with the blood vessel tissue, and firmly attached at the embolization site. SMMs retained their helical morphology without any fractures after four weeks of implantation, which indicates their mechanical stability and a lifespan of at least one month.

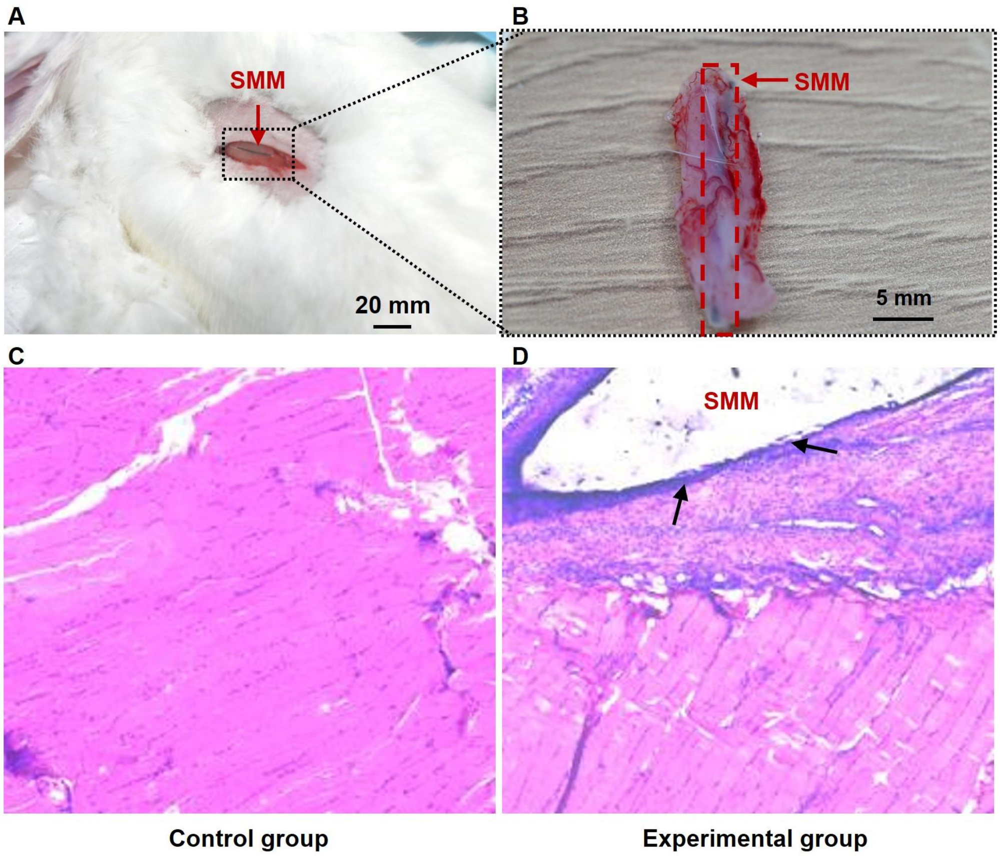

Fig.S14. A one-week SMM subcutaneous immune response test. (A) Implanted location: rabbit back. **(B)** Optical image of the SMM and surrounding tissue one week after subcutaneous implantation. **(C)** Control group: H&E staining of a rabbit with only an incision in the back and nothing implanted. **(D)** Experimental group: H&E staining result in vivo subcutaneous implantation using SMM.

Table S1. Limitations of the current minimally invasive endovascular operations for these diseases

Disease	Prevalence Rate	Symptoms	Current Treatment Methods	Limitations of Current Treatments	Potential Solutions (Our contribution)
Aneurysms (Brain, Aorta)	3-5% [42]	Headache, Bleeding	Endovascular repair, Surgical repair, Catheter embolization	Risk in catheter-based surgeries hard-to-reach some cases	Improvement in endovascular techniques, advancements in surgical methods
Brain Aneurysm	1-5% [42]	Headache, Dizziness, Vision Problems	Endovascular repair, Catheter embolization	Hard to reach in some cases, surgical risk	Improvement in endovascular techniques, research in minimally invasive surgeries
Liver Tumor	2-3% [43]	Abdominal Pain, Nausea, Fatigue	Surgical resection, Chemotherapy, Radiofrequency ablation, Transcatheter embolization	Not suitable for surgery in some cases, rich liver blood supply	Increase surgical precision, advancements in interventional radiology
Vascular Tumor	<1%	Visual Impairment, Bleeding	Interventional Radiology, Surgical Resection	Complex catheter-based surgeries, risks involved	Advancements in interventional techniques, research in minimally invasive surgeries
Bleeding Control	Varies	Bleeding, Anemia	Endovascular repair, Intervention, Surgery	Catheter-based solutions may not be possible in some cases	Improved interventional techniques, better bleeding control strategies
Varicose Veins	10-15% [46]	Venous dilation, Pain	Endovascular catheter treatment, Surgical removal	Hard to reach some cases, and recurrence possible	Improved catheter technology, better long-term management strategies
Acute Pulmonary Embolism	1-2% [47]	Shortness of Breath, Chest Pain	Anticoagulant therapy, Thrombolytic therapy, Catheter-directed thrombolysis	Risks in catheter-based surgeries, limited patient selection	Use of ultrasound guidance, improved catheter technology
Kidney Disease	8-10%	Reduced Kidney Function	Endovascular repair, Intervention, Surgery	Hard to reach some cases, complex anatomy	Better endovascular repair techniques, personalized treatment plans

Table S2. Parameters of typical temperature-sensitive shape memory effects and mechanical properties of various gels reported

Entry	Mechanism of the shape memory	Rf (%)	Rr (%)	Response time	Response temperature (℃)	Data sources
Shape memory, magnetically-response gels	Crosslinked structure	92.46%	82.79%	2 s	38	**Our work**
Shape memory Organogel	Crosslinked structure	86.5%	99.3%	30s	37	Adv. Mater. 2023, 35, 2308130.
Gels(Hydrophobic Cellulose Nanofibers)	Chemically and physically crosslinked structures	80.0%	95.8%	N/A	N/A	Polymers 2023, 15, 3547
Polymer(Shape memory polymer and self-made gel)	Glass transition temperatures	N/A	N/A	N/A	Formation temperature	Mater. Res. Express 2023, 2053, acdc06
Polymer(Gelatin and tannic acid)	Hydrogen bond between gelatin and tannic acid	N/A	95.8%	30 s	37	ACS Omega 2023, 1021, 6730
Biobased plasticizers from sesame oil (HSSO)	Low-temperature thermal actuation	5%	94.44%	150 s	60	RSC Adv. 2022, 12, 06862
Poly(AN-AAm-PEGDMA) hydrogels	Disassociation/association of dipole-dipole interactions and H-bondings	97.5	100	4 s	37	Adv. Funct. Mater. 2018, 28, 1705962
Poly(N-isopropylacrylamide) (PNIPAAm)	Thermoresponsive hydration and dehydration of the hydrogel network	N/A	N/A	N/A	32	Journal of Chem. Phys. 1979, 70, 1214-1218.
Bilayer of electrostatic spinning pNIPAM	Osmotic driven heterogeneous deswelling	N/A	N/A	N/A	40	Adv. Mater. 2015, 27, 4865.
Stearyl side chains crystalline	Disassociation/formation of hydrophobic crystalline	N/A	N/A	N/A	50	Nature 1995, 376, 219
n-Octadecyl acrylate reinforced hydrogel	Disassociation/formation of hydrophobic crystalline	N/A	N/A	60 s	50	Macromolecules 2014, 47, 6889.
n-Octadecyl acrylate reinforced hydrogel	Disassociation/formation of hydrophobic crystalline	N/A	N/A	15 s	43-48	Macromolecules 2013, 46, 3125.

Oligo(tetrahydrofuran) side chain hydrogel	Disassociation/formation of hydrophobic crystalline	83-98	84-93	N/A	24-28	Macromol. Symp. 2014, 345, 8.
Oligo(ω-pentadecalactone) side chain hydrogel	Disassociation/formation of hydrophobic crystalline	95	92		90	Macromol. Mater. Eng. 2012, 297, 1184.
Oligo(tetrahydrofuran) and Oligo(ω-pentadecalactone) side chains hydrogel	Disassociation/formation of hydrophobic crystalline	89-97	90	20 min	60 and 90	ACS Appl. Mater. Interfaces 2016, 8, 28068.
Poly(vinyl alcohol)-poly(ethylene glycol) double-network hydrogel	Disassociation/formation of PVA crystalline			15 s	90	Langmuir 2015, 31, 11709.
GO/gelatin/PAAM hydrogel	Disassociation and formation of triple-helices of gelatin	100	100	10-60 s	80	ACS Appl. Mater. Interfaces 2016, 8, 12384.
PBA-catechol alginate hydrogel	Disassociation and association of PBA-catechol bonds	100	100	13 min	25	Polym. Chem. 2016, 7, 5343.
4-Hydroxybutyl acrylate-co-NIPAM porous hydrogel	Osmotic driven heterogeneous deswelling	100	100	10 s	40	Adv. Funct. Mater. 2015, 25, 7272.
GO/P(AM-co-PEGMA)/CD hydrogel	Thermally triggered disruption and construction of topological structures			11 s	80	Carbohydr. Polym. 2017, 174, 804.
Oxidized cellulose nanofibers/polyacrylamide/gelatin hydrogels	Disassociation and formation of triple-helices of gelatin	60		30 s	90	Carbohydr. Polym. 2017, 171, 77.

Rf: shape fixity ratio Rr: shape recovery ratio
N/A: The data are not available in the data sources.

Table S3. Data of relative speed versus mass of some animals as well as soft robots and actuators.

Species	Relative speed (BL/s)	Body mass (g)	Reference
**Mammal**			
Elephas maximus	1.18	4000000	Alexander, R. M. J. Zool. 1977, 183, 125-146.
Equus zebra	7.35	300000	Alexander, R. M.; Langman, V. A.; Jayes, A. S. J. Zool. 1977, 183, 291-300.
Uromys caudimaculatus	16.6	1180	Alexander, R. M.; Langman, V. A.; Jayes, A. S. J. Zool. 1977, 183, 291-300.
Gazella subgutturosa	25.5	30000	Alexander, R. M.; Langman, V. A.; Jayes, A. S. J. Zool. 1977, 183, 291-300.
Urocyon cinereoargenteus	29.6	3700	Alexander, R. M.; Langman, V. A.; Jayes, A. S. J. Zool. 1977, 183, 291-300.
Cheetah	14.88~	44000	DOI: 10.1038/nature25479
Male Impala	9.86~	60000	DOI: 10.1038/nature25479
Male Lion	6.5~	200000	DOI: 10.1038/nature25479
Zebra	4.43	250000	DOI: 10.1038/nature25479
Horse:	7.52~	700000	Evans, J. W. (1990). The Horse. W. H. Freeman and Company.
Rats:	28.85	400	Whishaw, I. Q., & Kolb, B. (2004). The Behavior of the Laboratory Rat: A
Human	7.31	62000	Weyand, Peter G. et al. "Faster top running speeds are achieved with greater
Cat	25.39	4500	Case, L. P. (2003). The Cat: Its Behavior, Nutrition, and Health. Iowa State Press.
**Arthropod**			
Leptogenys schwabi	1.6	0.00868	DOI: 10.1111/j.1365-3032.1993.tb00610.x
Leptogenys nitida	3.41	0.00174	DOI: 10.1111/j.1365-3032.1993.tb00610.x
Eremobates marathoni	10	2	Bartholomew, G. A.; Lighton, J. R. B.; Louw, G. N. J. Comp. Physiol. B 1985,
Paratarsotomus macropalpis	323	0.000107	Doi:10.1242/jeb.128652
Formica fusca	26.9	0.006	Hurlbert, A. H.; Ballantyne, F.; Powell, S. Ecol. Entomol. 2008, 33, 144–154.
Dolomedes plantarius	37.5	1.5	Bartholomew, G. A.; Lighton, J. R. B.; Louw, G. N. J. Comp. Physiol. B 1985,
Onymacris plana	50	0.73	Bartholomew, G. A.; Lighton, J. R. B.; Louw, G. N. J. Comp. Physiol. B 1985,
Periplaneta americana	50	0.83	Full, R. J.; Tu, M. S. J. Exp. Biol. 1991, 156, 215–231.
Cataglyphis bombycina	74	0.01	Wittlinger, M.; Wehner, R.; Wolf, H. J. Exp. Biol. 2007, 210, 198–207.
Schizocosa ocreata	75	0.05	Amaya, C. C.; Klawinski, P. D.; Formanowicz, D. R. Am. Midl. Nat. 2001,
Cicindela eburneola	171	0.05	Kamoun, S.; Hogenhout, S. A. Coleopts. Bull. 1996, 50, 221–230.
Saharan silver ant	97	0.0035	Doi:10.1038/s41598-019-44901-0
Drosophila melanogaster	80	0.001	DOI:10.1016/j.cub.2005.08.020
Honeybee	593	0.09	DOI:10.1006/anbe.2000.1455

**Soft robot and actuator**			
SMMs	5.08	0.0027	**Our work**
Chemical	0.00118	0.0012	Maeda, S.; Hara, Y.; Sakai, T.; Yoshida, R.; Hashimoto, S. Adv. Mater. 2007, 19,
Hydrogel	0.003	0.052*	Morales, D.; Palleau, E.; Dickey, M. D.; Velev, O. D. Soft Matter 2014, 10, 1337–
Shape memory alloy	0.005	9	Kim, B.; Lee, M. G.; Lee, Y. P.; Kim, Y.; Lee, G. Sens. Actuators A Phys. 2006,
Humidity	0.011	0.036*	Ma, Y.; Zhang, Y.; Wu, B.; Sun, W.; Li, Z.; Sun, J. Angew. Chem. Int. Ed. 2011,
Ionic polymer	0.016	3.58*	Tomita, N.; Takagi, K.; Asaka, K. In Proceedings of the 2011 SICE Annual
Magnetic	0.029	0.0394*	Lu, H.; Zhang, M.; Yang, Y.; Huang, Q.; Fukuda, T.; Wang, Z.; Shen, Y. Nat.
Magnetic	0.039	63*	Kwok, S. W.; Morin, S. A.; Mosadegh, B.; So, J.-H.; Shepherd, R. F.; Martinez, R.
Dielectric elastomer	0.05	0.09*	Xu, L. et al. Bioinspir. Biomim. 2017, 12, 025003.
Dielectric elastomer	0.2	35*	Nguyen, C. T. et al. Sens. Actuators A Phys. 2017, 267, 505–516.
Humidity	0.24	0.035*	Shin, B. et al. Sci. Robot. 2018, 3, eaar2629.
Dielectric elastomer	0.69	42.5	Li, T. et al. Sci. Adv. 2017, 3, e1602045.
Dielectric elastomer	1	0.04*	Duduta, M.; Clarke, D. R.; Wood, R. J. In Proceedings of the 2017 IEEE
Shape memory alloy	1	2.4	Hoover, A. M.; Steltz, E.; Fearing, R. S. In Proceedings of the 2008 IEEE/RSJ
Shape memory alloy	5	5*	Lin, H.-T. et al. Bioinspir. Biomim. 2011, 6, 026007.
Magnetic	6.4	0.025*	IEEE: 2017; pp 22-26 January.
Piezoelectric	10.1	1.27*	Baisch, A. T. et al. Int. J. Rob. Res. 2014, 33, 1063–1082.
Magnetic	12	0.005*	Hu, W. et al. Nature 2018, 554, 81–85.
DC motor	15	16	IEEE: 2009; pp 11-15 October.
DC motor	27	30	IEEE: 2013; pp 6-10 May.
DC motor	47	54	Autumn, K. et al. Nature 2000, 405, 681–685.
Piezoelectric	20	0.024*	DOI:10.1126/scirobotics.aax1594
piezoelectric	7.7	1.66	DOI:10.1126/scirobotics.aax7906

~ Approximate value

* Mass of external power not included

Note: Most of these speeds are chosen from the maximum speeds and do not represent general speeds.

Table S4. Comparison of primary modern medical imaging. DSA(angiography), ultrasound, and Doppler were used in this study.

Criteria	Angiography	Ultrasound Imaging	Doppler Imaging	Endoscopic Imaging	CT Scan	Source
Invasiveness	Invasive	Non-invasive	Non-invasive	Invasive	Non-invasive	DOI: 10.3171/2013.2.FOCUS1324 DOI: 10.17816/KMJ2313
Diagnostic Quality	High	Moderate	Moderate	High	High	DOI: 10.3171/2013.2.FOCUS1324 DOI: 10.5455/aim.2014.22.160-163
Radiation Exposure	Yes	No	No	No	Yes	DOI: 10.3171/2013.2.FOCUS1324 DOI: 10.5114/aoms.2012.31624
Contrast Media Requirement	Required	Optional	Optional	Optional	Optional	DOI: 10.3171/2013.2.FOCUS1324 DOI: 10.5455/aim.2014.22.160-163
Time Consumption	More	Less	Less	More	Moderate	DOI: 10.3171/2013.2.FOCUS1324 DOI: 10.17816/KMJ2313
Cost	Expensive	Moderate	Moderate	Expensive	Expensive	DOI: 10.5455/aim.2014.22.160-163 DOI: 10.17816/KMJ2313
Assessment of Blood Flow	Excellent	Good	Excellent	Good	Good	DOI: 10.3171/2013.2.FOCUS1324 DOI: 10.5114/aoms.2012.31624
Operator Dependency	Moderate	High	High	Moderate	Low	DOI: 10.5455/aim.2014.22.160-163 DOI: 10.17816/KMJ2313
Complications	Possible	Fewer	Fewer	Possible	Fewer	DOI: 10.3171/2013.2.FOCUS1324 DOI: 10.5114/aoms.2012.31624
**Used in this work**	**Yes**	**Yes**	**Yes**	**NO**	**NO**	**This work**

Table S5. T-test of Masson-stained positive area of kidneys

Comparison	T	P
Left kidney (embolized kidney) in 4-week experimental group **vs.** 0-week control group	12.202	<0.001 (***)
Right kidney of the experimental group at 4 weeks **vs.** right kidney of the control group at 0 weeks	-1.153	0.2 (ns)
Comparison of own left embolized and right unembolized kidneys in 4-week experiment group	8.890	<0.001 (***)
Comparison of own left and right kidneys in 0-week control group	1.811	0.05 (ns)

Degrees of freedom: 8
Significance Levels:

Highly Significant (***): $P<0.001$

Significant (**): $0.001 \leq P<0.01$

Moderately Significant (*): $0.01 \leq P<0.05$

Not Significant (ns): $P \geq 0.05$

Supplementary Movies

Movie S1.

Deformation of the Shape-memory Magnetic Microrobot (SMM) in 38℃.

Movie S2.

Multimodal motion of the SMM.

Movie S3.

Adaptation of multimodal motion of the SMM in a 3D carotid modal.

Movie S4.

Path following of the SMM in open space.

Movie S5.

Long-distance locomotion of the SMM in a human-sized modal.

Movie S6.

Validation of the multistage embolization strategy in a live rabbit.

Movie S7.

Reverse blood flow locomotion of the SMM in a live rabbit.
